# Developing and Validating Machine Learning-Driven Risk Indices to Predict Patient Dropout During Referral, Evaluation, and Waitlisting for Kidney Transplant

**DOI:** 10.1111/ctr.70325

**Published:** 2025-09

**Authors:** Solaf Al Awadhi, Enshuo Hsu, Thomas B. H. Potter, Ioannis A. Kakadiaris, David A. Axelrod, Faith Parsons, Andrea M. Meinders, Victoria Cassell, Catherine Pulicken, Zulqarnain Javed, Paula K. Shireman, Stefano Casarin, A. L. Jonathan Gelfond, Amy D. Waterman

**Affiliations:** 1 Department of Surgery, Houston Methodist, Houston, Texas, USA; 2 Center for Health Data Science & Analytics, Houston Methodist, Houston, Texas, USA; 3 Department of Computer Science, University of Houston, Houston, Texas, USA; 4 Department of Surgery, University of Iowa, Iowa City, Iowa, USA; 5 Department of Cardiology, Center for Cardiovascular Computation and Precision Health, Houston Methodist Research Institute, Houston, Texas, USA; 6 Departments of Medical Physiology and Primary Care & Rural Medicine, Texas A&M College of Medicine, Bryan, Texas, USA; 7 Center for Precision Surgery, Houston Methodist Research Institute, Houston, Texas, USA; 8 LaSIE, UMR 7356 CNRS, La Rochelle Université, La Rochelle, France; 9 Department of Population Health, University of Texas Health Science Center, San Antonio, Texas, USA

**Keywords:** African American, disparities, evaluation, Hispanic, kidney transplant, machine learning, referral, waitlisting

## Abstract

**Background:**

Transplant is the optimal treatment for kidney failure; however, disparities in access persist. We developed and validated risk indices to predict early dropout at key stages of the transplant-seeking process not captured in national registries.

**Methods:**

We included patients referred for kidney transplant at Houston Methodist Hospital between June 2016, and November 2023. We collected demographic, clinical, patient- and contextual-level socioeconomic variables from electronic health records and publicly available census data. We used machine learning (ML) models to predict the characteristics of patients at higher risk of dropping out: (1) at referral (before starting evaluation), (2) in the process of evaluation (before waitlisting), and (3) during waitlisting (before receiving a transplant). Model performance was evaluated using AUROC.

**Results:**

Of 4133 referred patients, 46% did not attend their first transplant evaluation visit. Of 2414 patients who were medically eligible for transplant and started evaluation, 54% did not become waitlisted. Of 2457 waitlisted patients, 31% became inactive on the waitlist. Higher risk patients were consistently older, obese, and socioeconomically disadvantaged, with stage-specific differences: social factors—such as being single, unemployed, less educated, and living in high-deprivation areas—and African American race dominated at referral (AUROC 0.79); clinical comorbidities and both African American and Hispanic ethnicity were prominent at evaluation (AUROC 0.71); and Hispanic ethnicity, smoking, and digital exclusion were key drivers at waitlisting (AUROC 0.76).

**Conclusion:**

ML models effectively identified dropout risk at referral, evaluation, and waitlisting, enabling early identification of at-risk patients. Targeted interventions could reduce disparities, improve evaluation completion, and increase transplant access.

## 1 | Introduction

Kidney transplant (KT) is the treatment of choice for patients with chronic kidney disease (CKD) [1]. However, over 800,000 disproportionately minoritized patients with CKD in the US remain on chronic dialysis [2], despite evidence that KT extends survival, improves quality of life, and reduces long-term costs [3–6]. Pursuing KT requires patients to travel to transplant centers and complete a series of rigorous medical and psychosocial assessments before waitlisting [7]. Once waitlisted, patients must complete annual evaluations to maintain their candidacy, with wait times often ranging from 5 to 10 years [8]. Due to the national kidney shortage, transplant candidates without a willing living donor are currently more likely to die than to successfully receive a transplant, especially patients in larger urban centers with prolonged waiting periods and those from underserved communities that experience greater levels of social disadvantage [8]. The complexity and uncertainty of this process is daunting and some potential candidates quit before their evaluation is even completed.

Despite efforts to eliminate disparities in access to KT, African American (AA), Hispanic, and other historically underserved and socioeconomically disadvantaged subgroups are more likely than White patients to drop out or “derail” during the evaluation and waitlisting processes [9–11]. We previously developed an individual-level, single-score Kidney Transplant Derailers Index (KTDI) predicting the risk of transplant dropout, which highlighted demographic and racial disparities in access to KT [12]. However, similar to other studies assessing the risk of dropout [13–18], the KTDI was developed using data from a smaller population (733 adults), focusing on a limited set of socioeconomic status (SES) variables, and used traditional regression modeling to evaluate the impact of social determinants of health (SDOH) on waitlisting and living donor transplantation rate. Machine learning (ML) is an impactful emerging tool in medicine with the advantage of modeling nonlinear relations and accounting for all possible interactions and effect corrections between variables. ML models have provided accurate, comprehensive, and personalized risk indices in breast cancer and acute kidney injury [19–21]. However, ML has not previously been used to predict risk of dropout prior to transplantation due to the lack of sufficiently robust training data as patient information at these early stages is not captured in national transplant registries. In order to target resource interventions to reduce early drop out, we need to effectively identify higher risk candidates based on characteristics identifiable at the time of referral and initial in person evaluation.

Using a novel large data set from an urban transplant program serving a large multiethnic population, we developed three robust ML models which incorporated an expanded list of patient-and contextual-level SES variables to predict the risk of patient dropout at early stages of the KT-seeking process. We developed personalized risk indices identifying key patient characteristics associated with dropout at three key time points: (1) at referral before starting evaluation, (2) before completing evaluation, and (3) after becoming waitlisted.

## 2 | Materials and Methods

### 2.1 | Study Design and Participants

**FIGURE 1 |:**
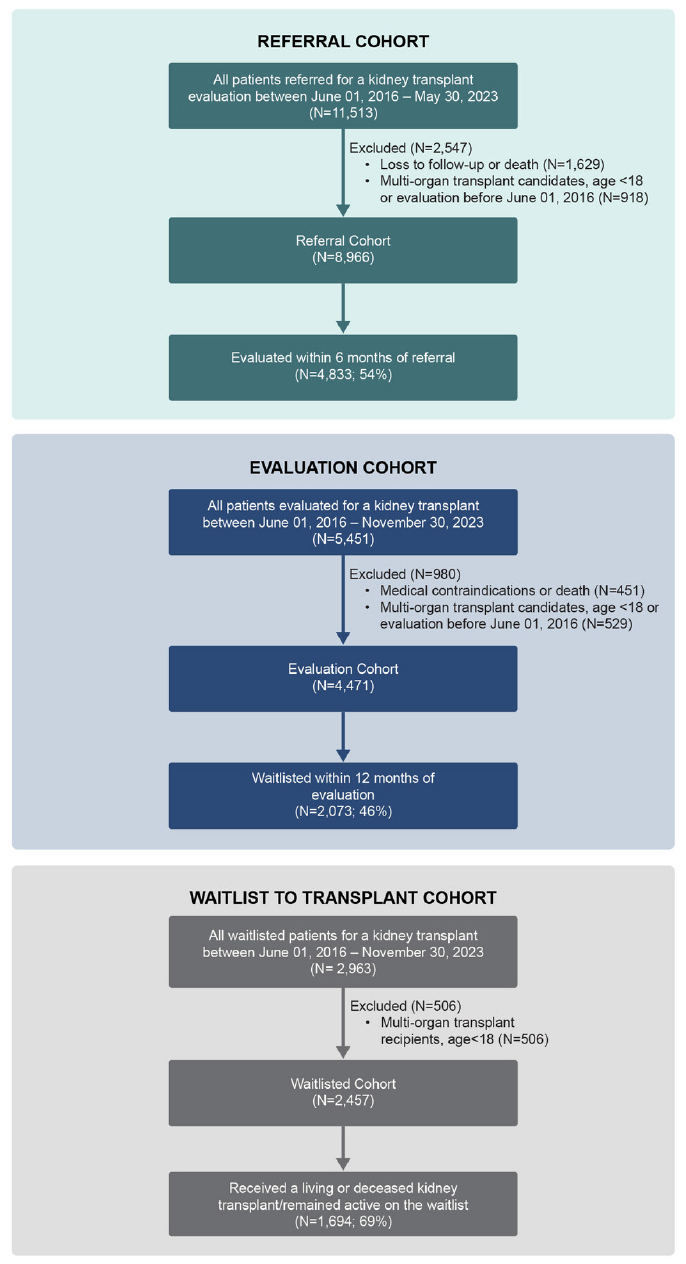
Consort diagram.

In this retrospective observational study, we obtained our participants’ sample by querying electronic health records (EHR) from the Houston Methodist Hospital’s (HM) KT population. Patients were divided into three cohorts for analysis: referral, evaluation, and waitlist (Figure 1). For all cohorts, we included patients ≥18 years old presenting for a KT at the HM J.C. Walter Jr. Transplant Center, and excluded patients referred for a multi-organ transplant.

For the referral cohort, we included CKD patients referred by a nephrologist or primary care physician to HM for KT evaluation between June 1, 2016 and May 30, 2023 (*N* = 8966). Referral was defined as a provider-submitted request that generated a transplant episode in the EHR. Dropout was defined as not presenting to a first evaluation visit at our center within 6 months of referral. Patients who were ineligible due to insurance (not covered under institutional contracts), could not be contacted, or died before evaluation were excluded. At referral, only limited data were available, as complete histories are obtained once evaluation begins; thus, exclusion for absolute contraindications could not be determined at this stage. To assess the impact of exclusions, we conducted a sensitivity analysis comparing patients who were included in the referral cohort with those who were excluded.

For the evaluation cohort, we included CKD patients who began KT evaluation between June 01, 2016 and November 30, 2022 (*N* = 4471). Patients who died or had absolute medical contraindications per Kidney Disease Improving Global Outcomes (KDIGO) guidelines [7] were excluded, as the study focuses on identifying potentially modifiable reasons for dropout. Dropout was defined as starting but not completing evaluation, excluding those denied for non-modifiable medical contraindications as recorded in the EHR.

For the waitlisted cohort, we included CKD patients waitlisted for KT between June 1, 2016 and November 30, 2022 (*N* = 2457). Dropout was defined as becoming inactive on the waitlist or not receiving a KT during this period, despite being medically and insurance eligible. The study was approved by the HM Institutional Review Board (IRB ID# MOD00005656) with a waiver of informed consent.

### 2.2 | Data Collection and Covariates

We created a transplant registry by integrating patient-level data from Houston Methodist EHRs and the Organ Procurement and Transplantation Network (OPTN). Using ArcGIS [22], we geocoded patient addresses and linked them to publicly available contextual data sources, including the CDC Social Vulnerability Index (SVI), the US Census Bureau’s American Community Survey, and the Neighborhood Atlas for the Area Deprivation Index (ADI). Demographic, clinical, and socioeconomic variables were extracted from the EHR, while contextual SES variables were derived from census-linked data using patient zip codes. Covariates were selected prior to analysis based on relevant literature and expert input from clinicians and researchers [17, 23–25]. A complete list of variables and sources is provided in Table S1.

### 2.3 | Outcomes

Primary outcomes were assessed by cohort:

#### Initiation of transplant evaluation:

defined as the proportion of referred patients who completed at least one initial evaluation visit at the transplant center (attendance at a transplant education class and being seen by at least one provider) within 6 months of referral, a timeframe selected based on research team consensus that patients who do not initiate evaluation within this period are unlikely to do so afterward.

#### Completion of transplant evaluation (waitlisting):

proportion of patients in the evaluation cohort who were activated on the deceased donor KT waiting list within 12 months of starting the evaluation process, a timeframe aligned with national guidelines that require evaluation to be up to date yearly.

#### Transplantation:

Proportion of patients in the waitlist cohort who received a living or deceased KT or remained active on the waitlist at the end of data collection.

### 2.4 | Statistical Analysis

We summarized continuous variables with means, standard deviations, medians, interquartile ranges, and categorical variables with frequencies and proportions. We compared means and proportions between groups with the Kruskal–Wallis test, one-way ANOVA, Chi-squared tests, or Fisher’s exact tests as appropriate. We used three classic ML algorithms to assess the associations between covariates and the outcomes studied. We followed the Transparent reporting of a multivariable prediction model for individual prognosis or diagnosis (TRIPOD) guidelines for reporting the development and validation of models [26] (Table S2).

### 2.5 | Model Development

#### Model training and validation:

**FIGURE 2 |:**
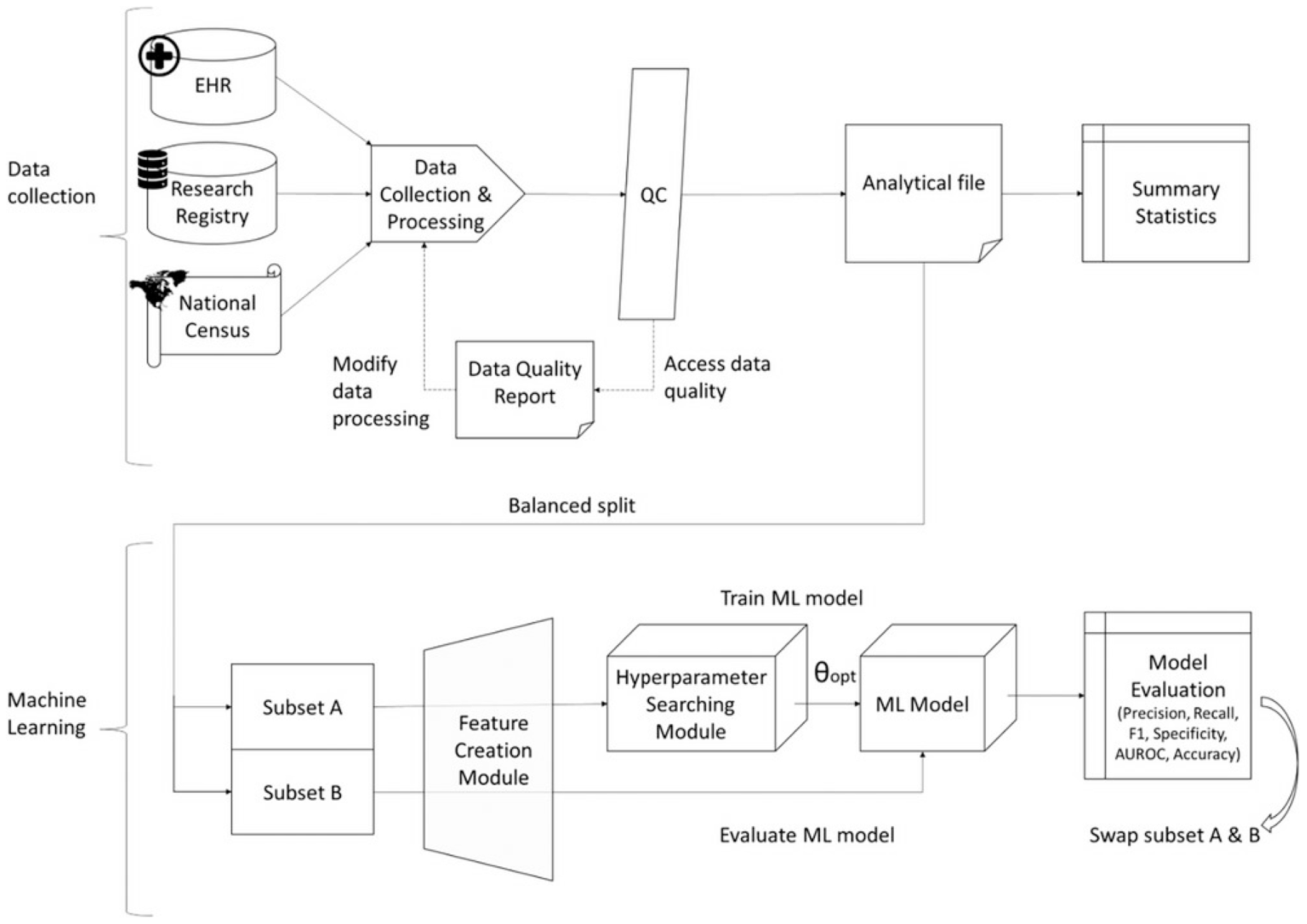
Stages of data collection and machine learning model development.

We followed a nested 2-fold cross-validation method by first splitting the dataset in half into equally-sized training and test subsets, balanced according to patient outcome, race/ethnicity, age, education, preferred language, diabetes and hypertension status, insurance status, distance from the hospital, and poverty as defined by the SVI. After initial model training and testing, data subsets were switched and the overall performance was determined as the average of the two separate runs [27]. To enhance robustness and reduce variability, model hyperparameters within each training set were optimized using nested 5-fold cross-validation grid search [27, 28]. Multiple feature engineering strategies, hyperparameter sets, and ML algorithms were explored for each cohort before training the final ML models (Figure 2). Data quality assessment methods are provided in the Supporting Information.

#### Feature engineering:

The collected data were pre-processed by categorizing and normalizing variables and treating missing values. We developed two feature engineering modules; in the first, denoted as “feature processor 0”, categorical variables were dummy coded (“one-hot encoding”), and continuous variables were categorized into predetermined buckets and dummy coded. Missing values were handled as a separate category (“missing”). In the second module, denoted as “feature processor 1”, categorical variables were dummy coded into nominal categories, which also allowed handling of missing values by turning them into zero-value vectors. Continuous variables were normalized by removing the mean and scaling to unit variance and missing values were imputed with the median value which was calculated using the training set.

#### Hyperparametric searching:

The hyperparameter searching was performed solely on the training sets. We performed a grid search with five-fold cross-validation. The ML model training used the hyperparameter set that yields the best area under the receiver operating characteristic curve (AUROC).

#### ML algorithms:

We tested the following classic ML models that are commonly used in clinical risk evaluation: [29] Support Vector Machines models (SVM), eXtreme Gradient Boosting (XGBoost), and Random Forest (RF). Using cross-validation within the training set, we performed a grid search to find the optional hyperparameters. The final model used was XGBoost for referral, and RF for evaluation and waitlisting (Table S3). We classified patients into low (0%–30%), middle (31%–60%), and high (61%–100%) dropout risk groups for interpretability. Cutoffs were based on the skewed probability distribution (skewness = 0.63; median = 30.3%; top quartile = 57.8%) and rounded to 30% and 60% as intuitive thresholds. These groups reflect the natural risk distribution in our cohort and were selected for descriptive comparison. The model itself remains valid across the full continuous range and the thresholds are intended only as adaptable descriptive strata, not fixed clinical cutoffs.

As a supplementary analysis to account for variable follow-up duration in the waitlist cohort, we performed a Cox proportional hazards analysis to assess baseline factors associated with dropout risk.

#### Performance assessment:

Performance for each ML algorithm and feature processor combination was assessed using AUROC as the primary metric for model selection, along with precision, recall, F1, specificity, and accuracy (Table S3).

Data were collected and processed using *Microsoft SQL server* and *Python 3.9.7*. Statistical analysis and ML were performed with Python *scikit-learn 1.3.2* and *xgboost 2.0.2*.

## 3 | Results

### 3.1 | Dropout at Referral Before Starting Evaluation: Referral Cohort

Among the 8966 patients who were referred for KT, of whom 36%, 28%, and 26% identified as AA, Hispanic, and White, respectively, 4133 (46.1%) dropped out within 6 months of referral to KT before ever starting evaluation (Table 1). Demographic characteristics differed by race/ethnicity for this cohort: there were fewer married AA patients compared to White and Hispanic patients (43% vs. 64% and 61%, *p* < 0.001, Table S4). More White patients reported being active or former smokers than AA and Hispanic patients (46% vs. 37% and 35%, *p* < 0.001). AA and Hispanic patients also had lower rates of private insurance compared to White patients (29% and 35% vs. 39%, *p* < 0.001).

Clinically, compared to White patients, AA and Hispanics had greater prevalence of hypertension (40% and 31% vs. 30%, *p* < 0.001) and diabetes (36% and 34% vs. 29%, *p* < 0.001), and spent a longer time on dialysis (564 and 423 vs. 297 days, *p* < 0.001). Fewer AA and Hispanic patients presented with possible living donors than White patients (8.5% and 13% vs. 19%, *p* < 0.001). Contextually, AA and Hispanic patients more frequently lived in geographical areas with a lower median annual household income than White patients ($48K and $55K vs. $78K, *p* < 0.001).

A sensitivity analysis comparing patients included in the referral cohort with those who were excluded showed no statistically significant differences in age (*p* = 0.1044) or sex (*p* = 0.253). This analysis was limited by the lack of additional variables available at the time of referral for excluded patients.

### 3.2 | ML Performance and Predictors of Dropout Before Starting Evaluation

**FIGURE 3 |:**
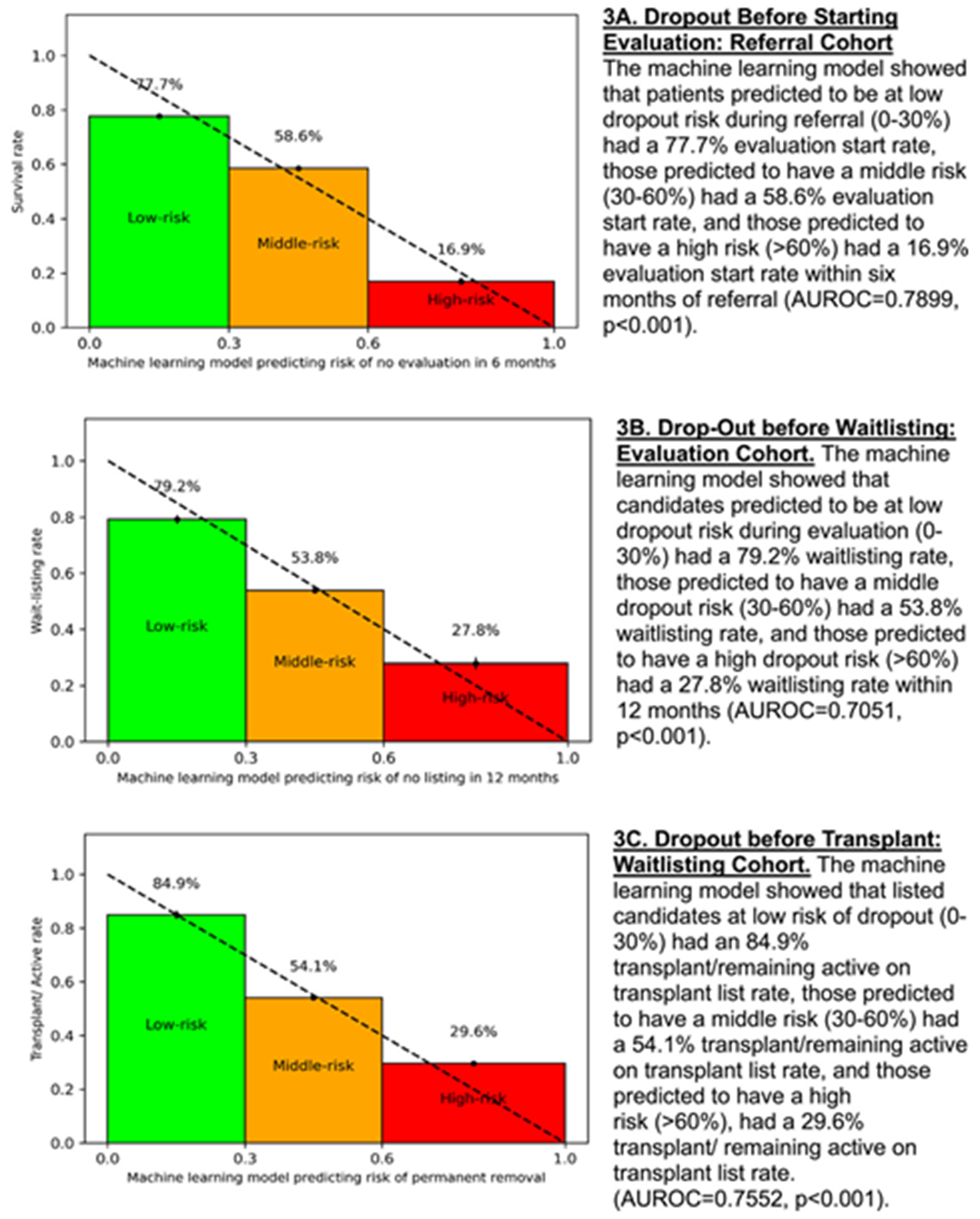
Machine LEARNING MODEL RISK INDEX PREDICTIOn Rate.

The ML model effectively predicted evaluation initiation within 6 months of referral, with rates of 77.7%, 58.6%, and 16.9% among low-, middle-, and high-risk groups, respectively (AUROC = 0.79, *p* < 0.001; Figure 3A, Table S3).

Several demographics, clinical, and contextual characteristics differed significantly across risk groups at referral (*p* values < 0.05). Median age increased with risk (54.7, 57.5, 58.3 years), and African American patients were more prevalent in the middle-and high-risk groups (42.4%, 41.9%) than in the low-risk group (26.3%), while the proportion of White patients declined by risk level (32.7%, 22.1%, 19.8%). Hispanic representation peaked in the middle-risk group (31.1%). Socioeconomic disadvantages generally rose with risk, including higher rates of single status, unemployment, lower education, and Medicare coverage. Obesity increased across risk groups (34.5%, 42.2%, 53.3%), while diabetes was most common in the middle-risk group (36.0%). Contextual disadvantage also followed this pattern, with higher ADI and SVI scores and reduced internet access observed among higher-risk patients (Table S5).

### 3.3 | Dropout Before Waitlisting: Evaluation Cohort

Among the 4471 candidates who were evaluated for KT, of whom 33%, 30%, and 28% identified as AA, Hispanic and White, respectively, 2398 (53.6%) dropped out during evaluation before becoming waitlisted (Table 1). Similarly to the referral cohort, fewer AA candidates were married than White and Hispanic patients (49% vs. 67% and 62%, *p* < 0.001), and more AA and Hispanic candidates were unemployed than White candidates (48%, 55% vs. 32%, *p* < 0.01; Table S6). Fewer AA and Hispanic candidates had private insurance than White patients (32% and 36% vs. 44%, *p* < 0.001).

Clinically, compared to White candidates, more AA and Hispanic candidates had comorbidities, including hypertension (54%, 43% vs. 39%, *p* < 0.01) and diabetes (53%, 62% vs. 46%, *p* < 0.01), and were on dialysis longer (446 days and 365 vs. 298 days, *p* < 0.001) and fewer had intended donors (17%, 22%, and 32%, *p* < 0.001). More AA candidates were obese (51% vs. 42% and 41%, *p* < 0.01) and compared to Hispanic and White candidates.

Contextually, more AA and Hispanic individuals lived within geographical areas with lower yearly household median incomes than Whites ($52K and $55K vs. $81K, *p* < 0.01).

### 3.4 | ML Performance and Predictors of Dropout Before Waitlisting

The ML model effectively predicted waitlisting within 12 months of evaluation start, with rates of 79.2%, 53.8%, and 27.8% among low-, middle-, and high-risk groups, respectively (AUROC = 0.71, *p* <0.001; Figure 3B, Table S3).

Several demographics, clinical, and contextual characteristics differed significantly across risk groups during evaluation (*p* values < 0.05). Median age increased with risk (46.9, 54.3, 60.0 years), and African American patients were more represented in higher-risk groups (16.7%, 27.2%, 44.9%), while White patient representation declined (48.8%, 31.0%, 18.4%). Hispanic patients were more common in the middle-and high-risk groups (30.9%, 31.6%) than in the low-risk group (16.3%). Socioeconomic disadvantages intensified across risk levels, including higher rates of single status, unemployment, lower education, and Medicare coverage, alongside a decline in private insurance. Clinical burden followed a similar trend, with increased rates of obesity (35.6%, 39.5%, 49.1%), diabetes (21.2%, 48.1%, 69.5%), and coronary artery disease (3.0%, 17.9%, 36.4%) in higher-risk groups. Contextual disadvantage also rose; lower median household costs, increased poverty (SVI), higher ADI, and reduced internet and computer access were observed (Table S7).

### 3.5 | Dropout Before Transplant: Waitlisted Cohort

Among the 2457 listed candidates, of whom 31%, 29%, and 33% identified as AA, Hispanic, and White, respectively, 763 (31.1%) did not receive a transplant over 7 years of follow-up or became inactive on the waitlist (Table 1). Similarly to the previous two cohorts, fewer AA listed candidates were married than White and Hispanic (54% vs. 71% and 66%, *p* < 0.001; Table S8). More AA and Hispanic listed candidates were unemployed compared to White (45%, 49% vs. 33%, *p* < 0.01).

Clinically, more AA and Hispanic listed candidates had comorbid conditions compared to White candidates, including hypertension (68% and 63% vs. 61%, *p* < 0.001), and diabetes (50% and 55% vs. 45%, *p* < 0.001) and fewer had intended donors (29% vs. 34% and 42%, *p* < 0.001). AA listed candidates were also more likely to be obese (49% vs. 39% and 39%, *p* < 0.001) than White and Hispanic.

Contextually, more AA and Hispanic candidates lived in areas where individuals had lower median annual household incomes compared to listed White patients ($55K and $56K vs. $83K, *p* < 0.001).

### 3.6 | ML Performance and Predictors of Dropout Before Receiving a KT

The ML model effectively predicted dropout after waitlisting, with 84.9%, 52.9%, and 32.1% of low-, middle-, and high-risk patients, respectively, remaining active on the waitlist or receiving a transplant by the end of data collection (AUROC = 0.76, *p* < 0.001; Figure 3C, Table S3).

Several demographics, clinical, and contextual characteristics differed significantly across risk groups during waitlisting (*p* values < 0.05). Median age rose with risk (49.0, 59.0, 65.3 years), and Hispanic patients were more prevalent in the middle-and high-risk groups (33.1%, 32.6%) than in the low-risk group (25.2%). Socioeconomic disadvantage increased with risk, including higher rates of retirement, lower education, and Medicare coverage, and lower employment rates. Clinical burden also intensified, with higher prevalence of obesity (33.3%, 42.8%, 54.1%), diabetes (27.7%, 67.1%, 95.3%), and coronary artery disease (13.0%, 39.4%, 62.8%). Contextual disadvantage worsened across groups, with increasing ADI scores and decreasing internet access and computer ownership (Table S9).

In the supplementary survival analysis (Table S10 and Figure S1), pulmonary hypertension was the only variable significantly associated with dropout risk (adjusted HR = 3.38, 95% CI 1.34–8.54).

## 4 | Discussion

To reduce disparities in access, transplant centers must identify patients likely to face greater challenges in progressing successfully and intervene early to assist them. Although enhanced support can benefit all patients, targeting resources to those most in need is vital in a resource-constrained environment. In this study, we developed and validated three ML-driven risk indices incorporating demographic, clinical, and patient and contextual level SES variables to identify patients at risk of early dropout during the transplant-seeking process at referral, evaluation and waitlisting—stages not captured in national transplant databases.

We found that between 31% and 54% of patients dropped out during referral, evaluation, and waitlisting. Across all three transplant stages, patients at higher risk of dropout consistently exhibited greater sociodemographic and clinical vulnerability. However, the degree and pattern of risk factors varied slightly by stage. At referral, social determinants such as race (higher proportions of African American patients), being single, unemployed, and having lower education were strongly associated with higher dropout risk. During evaluation, these disparities deepened, with older age, lower income, and comorbidities like diabetes and vascular disease becoming more prominent. By waitlisting, clinical burden, particularly diabetes (up to 95%) and cardiovascular disease, as well as structural disadvantage (e.g., highest ADI, lowest digital access) became dominant differentiators. Overall, while sociodemographic disparities were evident early, clinical and neighborhood-level barriers intensified in later stages of the transplant process.

Interventions that have shown promise in the broader transplant literature for supporting patients pursuing transplantation include frequent discussions with social workers, especially for patients who lack family support [30, 31], and peer mentoring, where previous kidney recipients share experiences to help overcome social and psychological barriers [32–35]. Tailored transplant education, culturally and linguistically adapted mobile health resources [36–40], direct home delivery of transplant education [10, 41], and involving patient’s social network in the learning process [32, 42] have improved knowledge, willingness, and pursuit of transplant [36–38, 43, 44]. Early discussions about insurance and financial aid can also reduce dropouts due to cost concerns [45] Targeted strategies—such as insurance navigation at referral, financial support during evaluation, and coverage advocacy at waitlisting—may reduce insurance-related disparities [46]. Although KDIGO guidelines recommend smoking cessation rather than exclusion [47], a US survey found 38% of centers consider active smoking a contraindication [48]. Access to smoking cessation programs helps more patients quit and progress to transplant [49]. However, these interventions have been tested as single-arm or qualitative studies, isolated components, or focused on different outcomes, highlighting the need for rigorous, stage-specific testing. Our study addresses this gap by introducing a systematic, data-driven method to identify high-risk patients across stages to enable targeted intervention development and evaluation.

At the health systems level, our findings align with the Increasing Organ Transplant Access (IOTA) Model mandated by the Centers for Medicare and Medicaid Services (CMS) which seeks to improve patient-centeredness and equity in KT access [50]. The CMS has also proposed “proportion of prevalent patients waitlisted” as a new quality metric to incentivize dialysis centers to increase referrals for KT evaluation [51, 52]. However, a lack of time, training, and resources among transplant educators in dialysis centers has limited their ability to fulfil this mandate [53, 54]. By targeting supplemental education and follow-up to high-risk patients earlier, barriers to waitlisting could be mitigated.

This study builds on previous work [12] by expanding the scope of variables analyzed, increasing the diversity and size of the study population, and applying more advanced methodology. Although both studies identify socioeconomic and demographic contributors to disparities in transplant access, this analysis is more comprehensive, using EHR-extracted clinical and social variables and contextual zip code–linked data instead of patient interviews. It also captures patients across multiple transplant stages—referral, evaluation, and waitlisting—including those who never attended an evaluation, offering a fuller picture of the transplant journey. In contrast to traditional logistic regression models, our use of ML accounts for complex, nonlinear interactions without prespecified assumptions and generates a single, intuitive risk index. This index integrates multiple factors into a unified, data-driven measure, improving usability in clinical settings. Although descriptive summaries of patient characteristics across risk groups provide useful contextual insights, they are observational and not intended for manual risk classification. Instead, the ML-derived risk index should be used for identifying risk. Importantly, because the model relies on structured EHR and publicly available contextual data, this approach improves scalability and generalizability across institutions, supporting broader implementation through strategies such as federated learning [55, 56].

Next steps include externally validating our models via federated learning across multiple transplant centers and convening a patient advisory board—comprised of higher risk patients identified by the risk models—to conduct meetings aimed at identifying potential modifiable barriers and facilitators. Building on these patient-informed insights, we will develop and rigorously evaluate targeted interventions to assess their impact on improving outcomes among higher risk populations across the different transplant stages. Ultimately, we aim to deliver a validated, clinically integrated risk algorithm that enables early identification of high-risk patients and guides the implementation of tailored, evidence-based interventions at each stage of the transplant process. It is essential to be aware of the potential risk of exacerbating inequities by intentionally or unintentionally focusing interventions on the patients deemed most likely to succeed rather than those most at risk necessitating careful ethical consideration when applying these risk indices to clinical decision-making [57, 58]. A set of recommendations outlining individual and system-level responsibilities, along with guidance on effective communication with vulnerable patients, will be needed [59].

The main limitations of the study include, first, its observational design, which prevents causal inferences, and the lack of external validation. Second, the accuracy of the exclusion criteria for medical and surgical contraindications, which rely on EHR reporting, should be further investigated to assess potential misclassification risks. Third, some variables (e.g., smoking history) had substantial missing data, reflecting common challenges in retrospective EHR data analyses, which we addressed by encoding missing categorical variables as a distinct category and imputing missing continuous variables with the median. Although this approach allowed retention of all individuals, median imputation reduces variability and may bias estimates; future studies could explore more robust approaches such as multiple imputation. Fourth, ~14% of referred patients were excluded due to loss to follow-up or death, introducing potential selection bias. Although our sensitivity analysis showed no age or sex differences, referrallevel data are inherently limited; complementary strategies such as prospective follow-up or linkage with referral sources could better characterize this group. Fifth, patients ineligible due to insurance type were also excluded, reflecting institutional policy; future work should examine this group and test interventions to reduce insurance-related barriers to transplant access. Finally, because our ML models do not account for varying times on the waitlist, we added a supplementary survival analysis to address time-to-event outcomes. This analysis identified few predictors, likely due to correlated covariates and non-linear relationships. As our primary goal was risk prediction rather than estimation of hazard over time, we used ML as the main analytic approach, which better handles high-dimensional data [19, 60, 61].

In conclusion, our models effectively identify patients at risk of dropout earlier in the transplant process by leveraging demographic, clinical, and socioeconomic data available in EHR to target interventions to reduce barriers and improve transplant access before dropout occurs. These risk indices can be integrated into clinical workflows to guide tailored solutions—such as education, peer support, and smoking cessation programs—developed in collaboration with clinicians, social workers, community partners, and informed by input from high-risk patients, ultimately enhancing equity and completion of transplant evaluation.

## Supplementary Material

Supplemental Material **Supporting Table 1:** Data Collection Covariates. **Supporting Table 2:** Transparent reporting of a multivariable prediction model for individual prognosis or diagnosis (TRIPOD): The TRIPOD statement. **Supplementary Methods:** Data Quality Assessment. **Supporting Table 3:** Machine Learning Configuration and Performance Metrics. **Supporting Table 4:** Differences in Patient Characteristics by Race at Referral. **Supporting Table 5:** Patient Characteristics Predicting Risk of Dropping Out at Referral. **Supporting Table 6:** Differences in Patient Characteristics by Race at Evaluation. **Supporting Table 7:** Patient Characteristics Predicting Risk of Dropping Out during Evaluation. **Supporting Table 8:** Differences in Patient Characteristics by Race at Waitlisting. **Supporting Table 9:** Patient Characteristics Predicting Waitlisting Outcomes.

Additional supporting information can be found online in the Supporting Information section.

## Figures and Tables

**TABLE 1 |:** Patient characteristics by cohort.

Patient characteristics		Referral cohort (N = 8966)	Missing data, N (%)	Evaluation Cohort (N = 4471)	Missing data, N (%)	Waitlisted Cohort (N = 2457)	Missing data, N (%)
**Demographics**							
Age (year); median (IQR)		56.7 (46.4–65.3)	0(0)	56.1 (45.3–64.3)	0(0)	54.8 (44.3,63.0)	0(0)
Sex; n (%)	Female	3576 (39.9)	0(0)	1801 (40.3)	0(0)	982 (40.0)	0(0)
Ethnicity/Race; n (%)	Hispanic	2548(28.4)	0(0)	1335 (29.9)	0(0)	710 (28.9)	0(0)
	Non-Hispanic AA	3218 (35.9)		1485 (33.2)		711 (31.4)	
	Non-Hispanic White	2298 (25.6)		1235 (27.6)		799 (32.5)	
	Other	902 (10.1)		416 (9.3)		237 (9.6)	
Marital status; n (%)	Married	4874 (55.9)	247 (3)	2673 (60.0)	13(0)	1593 (65.1)	11(0)
	Single	3845 (44.1)		1785 (40.0)		853 (34.9)	
Smoking; n (%)	Active/Former	2210 (38.0)	3155 (35)	1523 (34.8)	99(2)	693 (30.3)	225 (9)
	Never	3610 (62.0)		2849 (65.2)		1539 (69.0)	
	English	7787 (88.7)	191 (2)	3940 (88.1)	1(0)	2193 (89.3)	2(0)
Preferred language; n (%)	Spanish	828 (9.4)		437 (9.8)		217 (8.8)	
	Other^a^	160 (1.8)		93 (2.1)		45 (1.8)	
Employment; n (%)	Employed	2195 (26.3)	604 (7)	1319 (30.1)	82(2)	840 (35.0)	59(2)
	Unemployed	3986 (47.7)		1950 (44.4)		992 (41.4)	
	Retired	2181 (26.1)		1120 (25.5)		361 (24.7)	
Education; n (%)	Less than college	3042 (41.0)	1550 (17)	1619 (37.6)	170 (4)	863 (36.4)	89(4)
	College or higher	4374 (59.0)		2682 (62.4)		1505 (63.6)	
Insurance; n (%)	Medicare	5230 (58.3)	256 (2.9)	2705 (60.5)	2 (0.0)	1243 (50.6)	1 (0.0)
	Private	3003 (33.5)		1678 (37.5)		1178 (47.9)	
	Medicaid	241 (2.7)		51(1.1)		24(1.0)	
	Other/none	236 (2.6)		35 (0.8)		11 (0.5)	
**Clinical characteristics**							
BMI; n (%)	Underweight	153 (2.0)	1111(12)	68 (1.5)	23(1)	35(1.7)	357(7)
	Normal	1966 (25.0)		1064 (23.9)		508 (24.2)	
	Overweight	2462 (31.3)		1413 (31.8)		721 (34.3)	
	Obese	3274 (41.7)		1903 (42.8)		836 (39.8)	
Hypertension; n (%)		2959 (33.0)		2031 (45.4)		1562 (63.6)	0(0)
Diabetes; n (%)		2906 (32.4)	0(0)	2378 (53.2)		1211 (49.3)	0(0)
Coronary artery disease; n (%)		1663 (18.6)	0(0)	1085 (24.3)	0(0)	651 (26.5)	0(0)
Cerebrovascular accident; n (%)		735 (8.2)	0(0)	557 (12.5)	0(0)	235 (9.6)	0(0)
Peripheral vascular disease; n (%)		954 (10.6)	0(0)	786 (17.6)	0(0)	619 (25.2)	0(0)
Pulmonary hypertension: n (%)		369 (4.1)	0(0)	195 (4.4)	0(0)	134 (5.5)	0(0)
History of malignancy; n (%)		306 (3.4)	0(0)	252 (5.6)	0(0)	171 (7.0)	0(0)
Dialysis time (days); median (IQR)		431.0 (138.0–1195.8)	4248 (47)	366.0 (158.3–967.0)	2057 (46)	526.0 (285.0–1039.5)	1282 (52)
Dialysis type: n (%)	Hemodialysis	4666 (98.9)	4248 (47)	2385 (98.8)	2057 (46)	1155 (98.3)	0(0)
	Peritoneal dialysis	52(1.1)		29(1.2)		20(1.7)	
Patients with intended donor; n (%)		1157 (12.90)	0(0)	1044 (23.35)	0(0)	873 (35.5)	0(0)
Prior transplant; n (%)		211 (2.4)	0(0)	133 (3.0)	0(0)	108 (4.4)	0(0)
**Contextual-level characteristics**							
Monthly house cost ($): median (IQR)		1118.0 (887.0–1455.0)	101 (1)	1187.0 (898.0–1481.0)	18(0)	1199.0 (915.0–1505.0)	9(0)
Yearly household income ($); median (IQR)		60477.0 (44318.0–81993.0)	101 (1%)	63766.0 (46543.0–85444.0)	18 (1)		
Distance to health center; median, median mi (IQR)		21.5 (12.5–73.7)	0(0)	21.1 (12.6–55.6)	0(0)	21.6 (13.4–49.4)	0(0)
Household size; median (IQR)		2.9 (2.6–3.1)	90(1)	2.9 (2.6–3.1)	13(0)	2.9 (2.6–3.1)	7(0)
Commute time to health center; median % (IQR)	<30 min	26.9 (20.8–35.3)	85(1)	26.7 (20.5–34.2)	0(0)	26.7 (20.4–34.2)	6(0)
	30–45 min	28.7 (24.0–32.7)		28.7 (24.4–32.7)		28.7 (24.2–32.2)	
	>45 min	41.4 (33.4–47.8)		42.2 (34.5–48.6)		42.8 (34.6–49.2)	
Grandparents responsible for grandchildren; median % (IQR)		31.7 (21.9–44.6)	144 (2)	31.1 (21.3–41.8)	0(0)	30.4 (21.0–41.5)	13(1)
Own a vehicle; median % (IQR)		98.0 (96.3–98.9)	91(1)	98.3 (96.5–98.9)	13 (0)	98.3 (96.7–99.0)	0(0)
Telework; median % (IQR)		6.4 (3.8–9.7)	91(1)	6.9 (4.0–10.4)	0(0)	7.2(4.3–10.8)	7(0)
Internet in household; median % (IQR)		90.8 (83.7–95.4)	90(1)	91.5 (84.7–95.5)	13 (0)	92.0 (85.1–96.0)	7(0)
Computer in household, median % (IQR)		75.7 (64.4–86.9)	90(1)	78.1 (65.6–87.5)	13 (0)	80.0 (67.3–89.7)	7(0)
Smartphone in household; median % (IQR)		92.5 (87.4–95.5)	90(1)	92.9 (88.2–95.6)	13 (0)	93.3 (89.1–95.7)	7(0)
Plumbing facilities; mean median % (IQR)		98.8 (97.2–99.5)	88(1)	98.9 (97.2–99.5)	0(0)	98.9 (97.3–99.6)	7(0)
Census SSI; median % (IQR)		27.9 (16.4–39.1)	0(0)	26.9 (13.9–38.4)	0(0)	25.4 (12.2–36.5)	0(0)
SVI below poverty; median % (IQR)		23.1(12.7–36.3)	89(1)	21.9 (11.1–34.3)	14(0)	20.3 (10.1–32.3)	7(0)
SVI unemployment; median % (IQR)		2.7(1.6–4.1)	89(1)	25.7 (15.7–39.5)	14(0)	2.5 (1.5–3.8)	7(0)
SVI low income; median % (IQR)		9.2 (6.3–12.8)	89(1)	8.9 (6.1–12.6)	14(0)	8.6(5.9–12.0)	7(0)
State ADI, median (IQR)		6(3–8)	131 (1.5)	5(3–7)	31(0)	5(3–7)	12(0)
SVI single parent; median % (IQR)		2.6(1.4–4.1)	0(0)	2.4 (1.3–3.9)	14(0)	2.4 (1.2–3.7)	7(0)
SVI mobile homes; median % (IQR)		2.8 (0.0–3.0)	0(0)	21.9(11.1–34.3)	0(0)	0.3 (0.0–2.9)	0(0)
SVI crowding; median % (IQR)		1.3 (0.44–2.4)	0(0)	1.2 (0.4–2.4)	14(0)	1.1 (0.4–2.3)	7(0)
**Successful outcome**^b^ **n (%)**		4833 (53.9)	0(0)	2073 (46.4)	0(0)	1694 (69.0)	0(0)

*Note:* Some patients are common between the three groups (referred for evaluation, evaluated, and waitlisted).

Abbreviations: AA, African American; BMI, Body Mass Index; IQR, interquartile range; Mi, Miles; SBP, systolic blood pressure; SD, standard deviation; SVI, Social Vulnerability Index as census tract level.

a Top other preferred languages included Vietnamese, Arabic, Urdu, and Mandarin Chinese.

b Successful outcome per cohort: For referred patients the successful outcome is starting evaluation within 6 months of referral. For evaluated patients, the successful outcome is becoming waitlisted within 12 months of evaluation. For waitlisted patients, the successful outcome is receiving a living or deceased kidney transplant or remaining actively listed at time of data collection.

## Abbreviations:

AA: African American
ADI: area deprivation index
AUROC: area under the receiver operating characteristic curve
CDC: Centers for Disease Control and Prevention
CKD: chronic kidney disease
CMS: Centers for Medicare & Medicaid Services
EHR: Electronic health records
HM: Houston Methodist
IRB: institutional review board
KDIGO: kidney disease improving global outcomes
KT: kidney transplant
KTDI: kidney transplant derailers index
ML: machine learning
RF: random forest
SES: socioeconomic status
SDoH: social determinants of health
SVI: social vulnerability index
SVM: support vector machines models
TRIPOD: transparent reporting of a multivariable prediction model for individual prognosis or diagnosis
OPTN: Organ Procurement and Transplantation Network
XGBoost: extreme gradient boosting
