# Supplementary material for: Developing and Validating Machine Learning-Driven Risk Indices to Predict Patient Dropout During Referral, Evaluation, and Waitlisting for Kidney Transplant
Source: Clin Transplant. Author manuscript; Available in PMC 2026 Mar 9. (PMC12970565; doi:10.1111/ctr.70325)
Supplement: Supplemental Material — Supporting Table 1: Data Collection Covariates. Supporting Table 2: Transparent reporting of a multivariable prediction model for individual prognosis or diagnosis (TRIPOD): The TRIPOD statement. Supplementary Methods: Data Quality Assessment. Supporting Table 3: Machine Learning Configuration and Performance Metrics. Supporting Table 4: Differences in Patient Characteristics by Race at Referral. Supporting Table 5: Patient Characteristics Predicting Risk of Dropping Out at Referral. Supporting Table 6: Differences in Patient Characteristics by Race at Evaluation. Supporting Table 7: Patient Characteristics Predicting Risk of Dropping Out during Evaluation. Supporting Table 8: Differences in Patient Characteristics by Race at Waitlisting. Supporting Table 9: Patient Characteristics Predicting Waitlisting Outcomes. [file NIHMS2144457-supplement-Supplemental_Material.pdf]

Table S1. Data Collection Covariates. We queried the Houston Methodist Data Registry and linked it with census tract data to collect demographic, clinical, socio-economic, and contextual-level variables.

Dataset	Data Source	Factor	Variable*	Additional Details
HM Data	EHR	Demographics	Date of birth	Patient date of birth
			Sex	Patient self-reported sex (female/male)
			Race	Patient self-reported primary/secondary race
			Ethnicity	Patient's self-reported ethnicity
			Language	Patient's self-reported preferred languages
			Marital status	Patient's self-reported marital status
			Smoking	Patient's self-reported smoking status
		Socio-economic	Census block ID	Geocoded from patient home address
			Insurance coverage	Private, Medicare, Medicaid, Other/None
			Patient employment status	Patient self-reported employment status
			Patient highest education	Patient self-reported highest education
			Distance (access-level)	Distance between patient's address and the transplant center in miles
		Clinical	Height	Height closest to evaluation (cm)
			Weight	Weight closest to evaluation (kg)
			BMI	Calculated by weight in kilograms (kg) divided by the square of height in meters (m^2)
			Blood Pressure	Systolic & diastolic blood pressures closest to evaluation (mmHg)
			History of Malignancy	Any type using ICD-10 codes (C00-C09, D00-D09, Z85, Z86)
			Diabetes	Types I and II using ICD-10 codes (E08, E09, E10.[1-9], E11, E13)
			Cerebrovascular Accident	ICD codes (G45.(8-9), G46.(0-8), I60, I61, I62, I63, Z86.73)
			Hypertension	ICD codes (I00-I02, I13.[0-2], I15, I16)
			Coronary Artery Disease	ICD codes (I20-I25, Z95.1, Z95.5, Z98.61)
			Pulmonary Hypertension	ICD codes (I27)
			Symptoms of Peripheral Vascular Disease	ICD codes (I70.[2-9], Z95.820- Z95.828, Z98.62)
			Donor counts	Number of Intended kidney donors
			Days from dialysis to evaluation	Days from first dialysis to evaluation
			Type of dialysis	Type of dialysis the patient was on at evaluation (if any)
			Prior transplants	Prior all organ transplants
			Outcome	Started evaluation, Waitlisted (active/inactive), transplanted.
External Data		Contextual level	GeoID	Geographic information identifier on patient's address
			Census household income	Median yearly household income by race/ethnicity
			Census household size	Mean household size (Capita)
			Census Cost for Housing	Mean Monthly Housing cost

For Review Only

	US Census Bureau ACS 5-year Data (2016-2020)		Census Commute time	% of respondents with commute time <30 mins, 35-44 mins, ≥45 mins
			Census Grandparents responsible for grandchildren	% of respondents
			Census Telework/work from home (WFH)	% of respondents
			Census Vehicle	% of vehicle allocated
			Census Plumbing	% of respondents who have plumbing facilities
			Census Internet	% of respondents who have internet subscription in household
			Census Computer	% of respondents who have computer access in household
			Census Smartphone	% of respondents who have smartphones access in household
			Census Property ownership	% of respondents in occupied housing units who owned (not rent)
			Census SSI	% of respondents living in household with SSI, cash public assistance income, or Food Stamps/SNAP in the past 12 months
	CDC		SVI*** - Below poverty	% of respondents below 150% poverty estimate
			SVI*** - Unemployed	Ratio of Civilian (age 16+) unemployed estimate
			SVI*** - Low income	Ratio of Housing cost burdened occupied housing units with annual income less than $75,000 (30%+ of income spent on housing costs) estimate
			SVI*** - Single-parent	Ratio of Single-parent household with children under 18 estimates,
			SVI*** - Mobile homes	Ratio of Mobile homes estimate
			SVI*** - No vehicle	Ratio of Households with no vehicle available estimate
			SVI*** - Crowding house	Ratio of occupied housing units, more people than rooms estimate
	Neighborhood Atlas		ADI	State rank by census block (1-10)****

Abbreviations: ACS, American Community Survey; ADI, Area Deprivation Index; CDC, Centers for Disease Control and Prevention; EHR, Electronic Health Records, ESRD; End-stage renal disease; HM, Houston Methodist; ICD-10, International Classification of Diseases; SSI, Supplemental Security Income; SVI, Social Vulnerability Index; UNOS, United Network for Organ Sharing

*All the listed variables were included in the models except for date of birth, Days from dialysis to evaluation, type of dialysis and GeoID

** The intended or potential living donor variable is collected during the referral intake process, where nurses document patient-reported information in the EHR, often prior to the first evaluation visit.

***We evaluated the listed SVI components.

**** ADI data was analyzed categorically as follows: 0-3, 4-5, 8-9,

For Review Only

Table S2: Transparent reporting of a multivariable prediction model for individual prognosis or diagnosis (TRIPOD): The TRIPOD statement.

		Reporting Item	Page Number
**Title**			
	#1	Identify the study as developing and / or validating a multivariable prediction model, the target population, and the outcome to be predicted.	1
**Abstract**			
	#2	Provide a summary of objectives, study design, setting, participants, sample size, predictors, outcome, statistical analysis, results, and conclusions.	3
**Introduction**			
	#3a	Explain the medical context (including whether diagnostic or prognostic) and rationale for developing or validating the multivariable prediction model, including references to existing models.	4
	#3b	Specify the objectives, including whether the study describes the development or validation of the model or both.	5
**Methods**			
Source of data	#4a	Describe the study design or source of data (e.g., randomized trial, cohort, or registry data), separately for the development and validation data sets, if applicable.	5
Source of data	#4b	Specify the key study dates, including start of accrual; end of accrual; and, if applicable, end of follow-up.	5-7
Participants	#5a	Specify key elements of the study setting (e.g., primary care, secondary care, general population) including number and location of centres.	5-6
Participants	#5b	Describe eligibility criteria for participants.	5-6
Participants	#5c	Give details of treatments received, if relevant	5-7

For Review Only

Outcome	#6a	Clearly define the outcome that is predicted by the prediction model, including how and when assessed.	6-7
Outcome	#6b	Report any actions to blind assessment of the outcome to be predicted.	NA
Predictors	#7a	Clearly define all predictors used in developing or validating the multivariable prediction model, including how and when they were measured	6, Table S1
Predictors	#7b	Report any actions to blind assessment of predictors for the outcome and other predictors.	NA
Sample size	#8	Explain how the study size was arrived at.	5-6
Missing data	#9	Describe how missing data were handled (e.g., complete-case analysis, single imputation, multiple imputation) with details of any imputation method.	8
Statistical analysis methods	#10a	If you are developing a prediction model describe how predictors were handled in the analyses.	8
Statistical analysis methods	#10b	If you are developing a prediction model, specify type of model, all model-building procedures (including any predictor selection), and method for internal validation.	7-8
Statistical analysis methods	#10c	If you are validating a prediction model, describe how the predictions were calculated.	7-8
Statistical analysis methods	#10d	Specify all measures used to assess model performance and, if relevant, to compare multiple models.	9
Statistical analysis methods	#10e	If you are validating a prediction model, describe any model updating (e.g., recalibration) arising from the validation, if done	7-8
Risk groups	#11	Provide details on how risk groups were created, if done.	9
Development vs. validation	#12	For validation, identify any differences from the development data in setting, eligibility criteria, outcome, and predictors.	7-8
**Results**			

For Review Only

Participants	#13a	Describe the flow of participants through the study, including the number of participants with and without the outcome and, if applicable, a summary of the follow-up time. A diagram may be helpful.	Figure 1
Participants	#13b	Describe the characteristics of the participants (basic demographics, clinical features, available predictors), including the number of participants with missing data for predictors and outcome.	25
Participants	#13c	For validation, show a comparison with the development data of the distribution of important variables (demographics, predictors and outcome).	NA
Model development	#14a	If developing a model, specify the number of participants and outcome events in each analysis.	9-13
Model development	#14b	If developing a model, report the unadjusted association, if calculated between each candidate predictor and outcome.	NA
Model specification	#15a	If developing a model, present the full prediction model to allow predictions for individuals (i.e., all regression coefficients, and model intercept or baseline survival at a given time point).	
Model specification	#15b	If developing a prediction model, explain how to the use it.	7-9
Model performance	#16	Report performance measures (with CIs) for the prediction model.	Table S2
Model-updating	#17	If validating a model, report the results from any model updating, if done (i.e., model specification, model performance).	NA
**Discussion**			
Limitations	#18	Discuss any limitations of the study (such as nonrepresentative sample, few events per predictor, missing data).	16
Interpretation	#19a	For validation, discuss the results with reference to performance in the development data, and any other validation data	NA
Interpretation	#19b	Give an overall interpretation of the results, considering objectives, limitations, results from similar studies, and other relevant evidence.	13-16

For Review Only

Implications	#20	Discuss the potential clinical use of the model and implications for future research	16
**Other information**			
Supplementary information	#21	Provide information about the availability of supplementary resources, such as study protocol, Web calculator, and data sets.	18
Funding	#22	Give the source of funding and the role of the funders for the present study.	17

Note: The TRIPOD checklist is distributed under the terms of the Creative Commons Attribution License CC-BY.

For Review Only

Supplementary Methods: Data Quality Assurance

We ensured that the analytic data was fit for purpose by iteratively conducting code reviews, independent reprogramming of select variables, and data quality assessments. With code reviews, we evaluated scripts to validate that the calculation of variables was consistent with both the inclusion and exclusion criteria and data requirements for ML. We selected a subset of variables from the analytic dataset reproduced by an independent programmer and compared the corresponding values with those in the analytic dataset. We calculated the proportion of missing data and confirmed that data for commonly available variables in the EHR such as age, sex, ethnicity and race are at least 95% complete with valid values. Other variables with higher rates of missingness were included in the analysis, and a description of how they were handled in the analysis are included in the methodology. We also conducted a frequency distribution analysis, and when applicable, combined categories with too few counts into an ‘Other’ category (e.g. Native American and Pacific Islander comprised <1% of the population and were coded as ‘Other’ race). The frequency analysis enabled us to confirm that each categorical variable contained the correct list of values, and continuous variables are within the expected range of possible values.

For Review Only

Table S3. Machine Learning Configuration and Performance Metrics.

Machine Learning Configuration	Evaluation Metric (average of 2-folds)					
	**Precision**	**Recall**	**F1**	**Specificity**	**AUROC**	**Accuracy**
			**Referral Cohort**			
SVM with feature processor 0	0.72	**0.61**	0.66	0.78	0.77	0.71
SVM with feature processor 1	0.73	0.59	0.65	0.80	0.77	0.71
XGBoost with feature processor 0	0.70	0.59	0.64	0.78	0.76	0.70
**XGBoost with feature processor 1**	**0.75**	0.60	**0.67**	**0.83**	**0.79**	**0.72**
RF with feature processor 0	0.72	0.57	0.63	0.79	0.76	0.70
RF with feature processor 1	0.75	0.57	0.65	0.82	0.78	0.72
			**Evaluation Cohort**			
SVM with feature processor 0	0.66	0.72	0.69	0.55	0.70	0.65
SVM with feature processor 1	0.65	**0.77**	0.70	0.51	**0.71**	0.65
XGBoost with feature processor 0	0.65	0.69	0.67	0.56	0.68	0.63
XGBoost with feature processor 1	0.65	0.69	0.67	**0.57**	0.68	0.64
RF with feature processor 0	0.65	0.76	0.70	0.52	0.70	0.65
**RF with feature processor 1**	**0.66**	0.75	**0.70**	0.54	0.71	**0.66**
			**Waitlist to Transplant Cohort**			
SVM with feature processor 0	0.63	0.30	0.41	0.91	0.74	0.73
SVM with feature processor 1	0.65	0.31	0.41	0.92	0.75	**0.73**
XGBoost with feature processor 0	0.57	0.39	0.46	0.87	0.73	0.72
XGBoost with feature processor 1	0.57	0.41	0.48	0.86	0.74	0.72
RF with feature processor 0	0.67	0.23	0.34	0.94	0.74	0.72
**RF with feature processor 1**	**0.67**	0.26	0.37	0.94	**0.76**	0.73
SVM with feature processor 0 (balanced)	0.31	0.17	0.22	0.95	0.74	0.71
SVM with feature processor 1 (balanced)	0.62	0.33	0.42	0.90	0.75	0.73
XGBoost with feature processor 0 (balanced)	0.54	**0.54**	**0.54**	0.79	0.73	0.71

For Review Only

XGBoost with feature processor 1 (balanced)	0.56	0.49	0.52	0.82	0.74	0.72
RF with feature processor 0 (balanced)	0.69	0.15	0.25	**0.97**	0.74	0.72
RF with feature processor 1 (balanced)	0.64	0.20	0.31	0.95	0.75	0.72

Abbreviations: AUROC, Area under the Receiver Operating Characteristic; RF, Random Forest; SVM, Support Vector Machines; XGBoost, eXtreme Gradient Boosting.

Table S4.Differences in Patient Characteristics by Race at Referral

Patient Characteristics		*Overall, N=8966 (100%)*	*Overall Missing Data, N (%)*	*African American, N=3218 (35.9%)*	*Hispanic, N=2548 (28.4%)*	*White, N=2298 (25.6%)*	*Other/ Unknown, N=902 (10.1%)*	*P-value*
Demographics								
Age (year); median (IQR)		56.7 (46.4-65.3)	0 (0)	55.0 (45.0-63.9)	55.0 (44.9-63.0)	60.8 (50.7-67.9)	57.2 (46.6-66.0)	<0.001
Sex; n (%)	Female	3576 (39.9)	0 (0)	1454 (45.2)	980 (38.5)	827 (36.0)	315 (34.9)	<0.001
Marital Status; n (%)	Married	4874 (55.9)	247 (3)	1375 (43.0)	1544 (61.1)	1446 (63.6)	509 (70.4)	<0.001
	Single	3845 (44.1)		1822 (57.0)	983 (38.9)	826 (36.4)	214 (29.6)	
Smoking; n (%)	Active/Former	2210 (38.0)	3155 (35)	795 (36.8)	575 (34.9)	709 (45.5)	131 (29.4)	<0.001
	Never	3601 (62.0)		1365 (63.2)	1072 (65.1)	850 (54.5)	314 (70.6)	
Preferred Language; n (%)	English	7787 (88.7)	191 (2)	3197 (99.8)	1726 (68.0)	2248 (98.3)	616 (82.8)	<0.001
	Spanish	828 (9.4)		1 (0.0)	810 (31.9)	10 (0.4)	7 (0.9)	
	Other	160 (1.8)		6 (0.2)	4 (0.2)	29 (1.3)	121 (16.3)	
Employment; n (%)	Employed	2195 (26.3)	604 (7)	729 (23.7)	610 (25.0)	637 (29.3)	219 (32.6)	<0.001
	Unemployed	3986 (47.7)		1597 (51.9)	1387 (56.9)	755 (34.7)	247 (36.8)	
	Retired	2181 (26.1)		752 (24.4)	439 (18.0)	785 (36.1)	205 (30.6)	
Education; n (%)	Less than college	3042 (41.0)	1550 (17)	969 (36.1)	1316 (61.1)	579 (29.4)	178 (29.3)	<0.001
	College or higher	4374 (59.0)		1718 (63.9)	839 (38.9)	1388 (70.6)	429 (70.7)	
Insurance; n (%)	Medicare	5230 (60.1)	256 (2.9)	2075 (65.4)	1512 (60.1)	1265 (56.0)	378 (49.4)	<0.001
	Medicaid	236 (2.7)		121 (3.8)	58 (2.3)	38 (1.7)	19 (2.5)	
	Private	3003 (34.5)		906 (28.6)	883 (35.1)	889 (39.4)	325 (42.5)	
	Other/None	241 (2.8)		69 (2.2)	63 (2.5)	66 (2.9)	43 (5.6)	
Clinical Characteristics								
BMI; n (%)	Underweight	153 (2.0)	1111 (12)	51 (1.8)	30 (1.3)	50 (2.4)	22 (3.2)	<0.001
	Normal	1966 (25.0)		634 (22.2)	531 (23.5)	526 (25.6)	275 (40.4)	
	Overweight	2462 (31.3)		792 (27.7)	760 (33.6)	685 (33.3)	225 (33.1)	
	Obese	3274 (41.7)		1379 (48.3)	941 (41.6)	796 (38.7)	158 (23.2)	
Hypertension; n (%)		2959 (33.0)	0 (0)	1278 (39.7)	796 (31.2)	696 (30.3)	189 (21.0)	<0.001
SBP; median (IQR)		140.0 (124.0-157.0)	6009 (67)	140.0 (123.0-158.0)	143.5 (127.0-	136.0	141.0 (128.0-	<0.001

					162.0)	(120.0-152.0)	157.0)	
DPB, median (IQR)		75.0 (66.0-84.0)	6009 (67)	76.0 (68.0-86.0)	76.0 (67.0-84.0)	73.0 (64.0-82.0)	74.0 (65.0-85.0)	<0.001
Diabetes; n (%)		2906 (32.4)	0 (0)	1113 (34.4)	923 (36.2)	663 (28.9)	207 (23.0)	<0.001
Coronary Artery Disease; n (%)		1663 (18.6)	0 (0)	637 (19.8)	442 (17.3)	476 (20.7)	108 (12.0)	<0.001
Cerebrovascular Accident; n (%)		735 (8.2)	0 (0)	334 (10.4)	180 (7.1)	171 (7.4)	50 (5.5)	<0.001
Peripheral Vascular Disease; n (%)		954 (10.6)	0 (0)	415 (12.9)	253 (9.9)	239 (10.4)	47 (5.2)	<0.001
Pulmonary Hypertension; n (%)		369 (4.1)	0 (0)	155 (4.8)	88 (3.5)	107 (4.7)	19 (2.1)	<0.001
History of Malignancy; n (%)		306 (3.4)	0 (0)	112 (3.5)	53 (2.1)	128 (5.6)	13 (1.4)	<0.001
Dialysis Time (days); median (IQR)		431.0 (138.0-1195.8)	4248 (47)	564.0 (158.0-1570.8)	422.50 (143.0-1093.8)	297.0 (105.5-734.0)	351.0 (111.0-1050.0)	<0.001
Dialysis Type; n (%)	Hemodialysis	4666 (98.9)	4248 (47)	1959 (99.2)	1483 (98.3)	903 (99.1)	321 (98.8)	0.076
	Peritoneal Dialysis	52 (1.1)		15 (0.8)	25 (1.7)	8 (0.9)	4 (1.2)	
Prior Transplant; n (%)		211 (2.4)	0 (0)	36 (1.1)	38 (1.5)	124 (5.4)	13 (1.4)	<0.001
Intended donor; n (%)		1157 (12.9)	0 (0)	272 (8.5)	322 (12.6)	443 (19.3)	120 (13.3)	<0.001
Contextual-level Characteristics								
Distance to Transplant Center (Mi); median (IQR)		21.5 (12.5-73.7)	0 (0)	18.1 (10.7-42.6)	20.0 (11.5-47.1)	35.0 (20.0-100.2)	20.6 (14.2-102.2)	<0.001
Yearly Household Income ($); median (IQR)		60477.0 (44318.0-81993.0)	101 (1%)	48070.0 (35137.0-68925.0)	54516.0 (45410.0-69523.0)	78197.0 (60000.0-101581.0)	84306.0 (57256.0-112111.5)	<0.001
Household size; median (IQR)		2.9 (2.6-3.1)	90 (1)	2.9 (2.6-3.1)	3.0 (2.7-3.2)	2.8 (2.6-3.0)	3.0 (2.6-3.2)	<0.001
Monthly House cost ($); median (IQR)		1118.0 (887.0-1455.0)	100 (1)	1089.0 (892.0-1338.0)	1044.0 (862.0-1297.0)	1188.0 (880.3-1511.0)	1321.0 (1025.0-1724.0)	<0.001
Commute Time to Health Center; median % (IQR)	<30 mins	26.9 (20.8-35.3)	85 (1)	26.7 (22.5-34.0)	27.8 (22.3-35.6)	27.4 (20.2-38.0)	25.8 (20.3-32.0)	<0.001
	30-45 mins	28.7 (24.0-32.7)		29.8 (26.6-33.8)	28.9 (24.4-32.6)	26.8 (19.8-30.6)	28.7 (25.7-32.5)	<0.001
	>45 mins	41.4		41.2	41.0	42.3	44.5	<0.001

For Review Only

		(33.4-47.8)		(34.6-45.9)	(33.1-45.8)	(32.2-51.3)	(36.5-51.1)	
Grandparents Responsible for Grandchildren; median % (IQR)		31.7 (21.9-44.6)	144 (2)	32.4 (22.4-46.3)	31.5 (22.4-39.9)	33.3 (21.7-45.5)	29.0 (19.5-39.4)	<0.001
Telework; median % (IQR)		6.4 (3.8-9.7)	91 (1)	5.7 (3.53-8.8)	5.9 (3.8-8.5)	7.4 (4.1-11.2)	8.5 (5.40-12.6)	<0.001
Own a Vehicle; median % (IQR)		98.0 (96.3-98.9)	91 (1)	97.7 (95.8-98.8)	97.9 (96.2-98.8)	98.5 (97.4-99.1)	98.4 (96.7-99.0)	<0.001
Internet in Household; median % (IQR)		90.8 (83.7-95.4)	90 (1)	89.3 (82.7-94.5)	89.3 (82.3-94.3)	92.4 (86.6-96.5)	94.3 (88.2-97.2)	<0.001
Computer in Household; median % (IQR)		75.7 (64.4-86.9)	90 (1)	74.2 (61.8-85.2)	71.0 (60.3-83.6)	81.1 (69.9-90.6)	84.6 (71.5-92.5)	<0.001
Smartphone in Household; median % (IQR)		92.5 (87.4-95.5)	90 (1)	92.2 (86.6-95.2)	91.0 (87.0-94.7)	93.0 (89.1-95.7)	94.3 (90.5-96.7)	<0.001
Plumbing Facilities; mean median % (IQR)		98.8 (97.2-99.5)	88 (1)	98.7 (97.0-99.5)	98.7 (97.2-99.4)	98.7 (96.7-99.5)	99.3 (98.4-99.7)	<0.001
Census SSI; median % (IQR)		27.9 (16.4-39.1)	0 (0)	31.7 (19.8-42.5)	31.8 (20.2, 40.4)	20.3 (11.1-31.7)	20.1 (10.8-35.4)	<0.001
SVI Below Poverty; median % (IQR)		23.1 (12.7-36.3)	89 (1)	26.6 (15.5-40.9)	28.1 (17.1-39.8)	16.8 (8.5-26.3)	17.2 (8.1-28.9)	<0.001
SVI Unemployment; median % (IQR)		2.7 (1.6-4.1)	89 (1)	3.1 (1.8-4.4)	2.6 (1.7-4.0)	2.3 (1.3-3.6)	2.6 (1.6-4.0)	<0.001
SVI Low Income; median % (IQR)		9.2 (6.3-12.8)	89 (1)	10.5 (7.6-14.3)	9.1 (6.6-12.3)	7.8 (5.4-10.7)	8.2 (5.4-12.0)	<0.001
SVI Single Parent; median % (IQR)		2.6 (1.4-4.1)	0 (0)	3.1 (1.7-4.8)	2.7 (1.6-4.1)	1.9 (1.0-3.2)	2.1 (1.1-3.6)	<0.001
SVI Mobile Homes; median % (IQR)		2.8 (0.0-3.0)	0 (0)	0.1 (0.0-1.7)	0.4 (0.0-3.1)	0.8 (0.0, 6.6)	0.0 (0.0-1.1)	<0.001
SVI Crowding; median % (IQR)		1.3 (0.44-2.4)	0 (0)	1.4 (0.54-2.4)	1.9 (0.86-3.0)	0.8 (0.2- 1.7)	0.9 (0.3-2.0)	<0.001
Outcome	Started evaluation within 6 months of referral	4833 (53.9)	0 (0)	1556 (48.4)	1446 (56.8)	1387 (60.4)	444 (49.2)	<0.001

Abbreviations: BMI, Body Mass Index; DBP, diastolic blood pressure; IQR, interquartile range; Mi, Mile; SBP, systolic blood pressure; SD, standard deviation; SSI, Supplemental Security Income; SVI, Social Vulnerability Index

Table S5. Patient Characteristics Predicting Risk of Dropping Out at Referral. This table shows the characteristics of patients at low, middle, and high risk of dropout at referral before initiating the evaluation process for a kidney transplant.

**Patient Characteristics**		**Overall, N=4483 (100%)**	**Overall Missing Data, N (%)**	**Low Risk, N=1777 (40%)**	**Middle Risk, N= 1432 (32%)**	**High Risk, N=1274 (28%)**	**P-value**
**Demographics**							
Age (year); median (IQR)		56.7 (46.4-65.3)	0 (0)	54.7 (44.5-64.0)	57.5 (47.9-65.3)	58.3 (47.8-67.9)	<0.001
Sex; n (%)	Female	1812 (40.4)	0 (0)	674 (37.9)	605 (42.2)	533 (41.8)	0.016
Race/Ethnicity; n (%)	Hispanic	1274 (28.4)	0 (0)	512 (28.8)	445 (31.1)	317 (24.9)	<0.001
	AA	1609 (35.9)		468 (26.3)	607 (42.4)	534 (41.9)	
	White	1149 (25.6)		581 (32.7)	316 (22.1)	252 (19.8)	
	Other/Unknown	451 (10.1)		216 (12.2)	64 (4.5)	171 (13.4)	
Marital Status; n (%)	Married	2441 (55.9)	119 (3)	1197 (67.5)	720 (50.5)	524 (45.0)	<0.001
	Single	1923 (44.1)		577 (32.5)	705 (49.5)	641 (55.0)	
Smoking; n (%)	Active or Former	1123 (38.3)	1552 (35)	412 (30.2)	404 (42.4)	307 (50.1)	<0.001
	Never or Unknown	1808 (61.7)		954 (69.8)	548 (57.6)	306 (49.9)	
Preferred Language; n (%)	English	3893 (88.7)	96 (2)	1586 (89.3)	1265 (88.5)	1042 (88.2)	0.596
	Spanish	413 (9.4)		155 (8.7)	142 (9.9)	116 (9.8)	
	Other	81 (1.8)		35 (2.0)	22 (1.5)	24 (2.0)	
Employment; n (%)	Employed	1096 (26.1)	291 (7)	753 (43.2)	185 (13.2)	158 (15.1)	<0.001
	Unemployed	1988 (47.4)		561 (32.2)	858 (61.2)	569 (54.3)	
	Retired	1108 (26.4)		428 (24.6)	360 (25.7)	320 (30.6)	
Education; n (%)	Less than college	1520 (41.0)	775 (17)	529 (29.8)	698 (49.2)	293 (57.1)	<0.001
	College or more	2188 (59.0)		1248 (70.2)	720 (50.8)	220 (42.9)	
Insurance; n (%)	Medicare	2584 (59.3)	129 (3)	768 (43.2)	1074 (75.1)	742 (64.7)	<0.001
	Medicaid	131 (3.0)		9 (0.5)	33 (2.3)	89 (7.8)	
	Private	1523 (35.0)		981 (55.2)	298 (20.8)	244 (21.3)	
	Other/None	116 (2.7)		19 (1.1)	25 (1.7)	72 (6.3)	
**Clinical Characteristics**							
BMI; n (%)	Underweight	86 (2.2)	535 (12)	45 (2.6)	23 (1.7)	18 (2.0)	<0.001
	Normal	982 (24.9)		464 (27.1)	310 (23.2)	208 (23.0)	
	Overweight	1245 (31.5)		611 (35.7)	438 (32.8)	196 (21.7)	

For Peer Review Only

	Obese	1635 (41.4)		590 (34.5)	564 (42.2)	481 (53.3)	
Hypertension; n (%)		1458 (32.5)	0 (0)	590 (33.2)	467 (32.6)	401 (31.5)	0.603
Diabetes; n (%)		1406 (31.4)	0 (0)	520 (29.3)	515 (36.0)	371 (29.1)	<0.001
Coronary Artery Disease; n (%)		825 (18.4)	0 (0)	290 (16.3)	278 (19.4)	257 (20.2)	0.012
Cerebrovascular Accident; n (%)		349 (7.8)	0 (0)	123 (6.9)	125 (8.7)	101 (7.9)	0.158
Peripheral Vascular Disease; n (%)		464 (10.4)	0 (0)	107 (6.0)	196 (13.7)	161 (12.6)	<0.001
Pulmonary Hypertension; n (%)		168 (3.7)	0 (0)	51 (2.9)	54 (3.8)	63 (4.9)	0.012
History of Malignancy; n (%)		147 (3.3)	0 (0)	59 (3.3)	47 (3.3)	41 (3.2)	0.988
**Contextual-level Characteristics**							
Distance to Transplant Center (Mi); median (IQR)		21.8 (12.6-77.3)	0 (0)	21.9 (14.3-43.9)	21.5 (11.8-84.6)	21.8 (11.0-90.3)	0.922
Monthly Household Cost; median (IQR)		1103.0 (885.0-1455.0)	54 (1)	1238.0 (985.0-1594.0)	1026.0 (851.0-1297.0)	997.0 (789.0-1269.0)	<0.001
Commute Time to Health Center; median (IQR)	<30 mins	26.9 (20.7-35.3)	45 (1)	25.8 (19.8-31.6)	28.8 (23.1-39.0)	28.7 (22.9-39.0)	<0.001
Commute Time to Health Center; median (IQR)	30-45 mins	28.6 (23.6-32.6)		28.5 (24.5-32.4)	28.6 (23.2-32.5)	28.7 (22.6-33.7)	0.561
Commute Time to Health Center; median (IQR)	>45mins	41.4 (33.3-48.4)		44.8 (37.9-52.0)	40.4 (30.6-45.5)	39.3 (30.6-45.7)	<0.001
Grandparents Responsible for Grandchildren; median (IQR)		31.9 (21.8-44.7)	75 (2)	29.0 (19.9-39.1)	33.3 (24.4-46.2)	34.3 (24.1-48.8)	<0.001
Telework; median (IQR)		6.4 (3.8-9.6)	48 (1)	7.7 (4.9-11.1)	5.7 (3.5-8.7)	5.5 (3.4-8.5)	<0.001
Own a Vehicle; median (IQR)		98.0 (96.2-98.9)	48 (1)	98.4 (97.0-99.0)	97.8 (95.9-98.9)	97.6 (95.6-98.7)	<0.001
Internet Subscription in Household; median (IQR)		90.6 (83.4-95.4)	47 (1)	92.7 (86.6-96.6)	89.2 (82.7-94.3)	87.6 (81.9-93.5)	<0.001
Computer in Household, median (IQR)		75.4 (64.1-86.9)	47 (1)	83.0 (69.0-90.9)	72.9 (61.8-84.3)	70.9 (60.7-83.1)	<0.001
Smartphone in Household; median (IQR)		92.4 (87.3-95.5)	47 (1)	93.8 (89.6-96.2)	91.4 (86.4-94.7)	90.1 (85.2-94.1)	<0.001
Plumbing Facilities; median (IQR)		98.7 (97.1-99.5)	46 (1)	99.1 (97.7-99.7)	98.6 (96.7-99.4)	98.4 (96.5-99.3)	<0.001
SVI Below Poverty; median (IQR)		23.3 (12.7-36.7)	48 (1)	18.8 (9.5-31.1)	25.1 (15.4-39.0)	27.9 (17.2-41.9)	<0.001

For Review Only

SVI Unemployment; median (IQR)		2.7 (1.6-4.1)	48 (1)	2.5 (1.6-3.8)	2.6 (1.6-4.1)	3.1 (1.8-4.4)	<0.001
SVI Low Income; median (IQR)		9.1 (6.3-12.9)	48 (1)	8.1 (5.7-11.7)	9.8 (6.8-13.2)	9.9 (6.9-13.8)	<0.001
SVI Single Parent; median (IQR)		2.6 (1.4-4.1)	48 (1)	2.3 (1.2-3.6)	2.7 (1.5-4.3)	2.9 (1.6-4.4)	<0.001
SVI Mobile Homes; median (IQR)		0.3 (0.0-3.1)	48 (1)	0.3 (0.0-3.4)	0.2 (0.0-2.7)	0.3 (0.0-3.2)	0.519
SVI Crowding; median (IQR)		1.3 (0.4-2.4)	48 (1)	1.1 (0.3-2.2)	1.4 (0.5-2.5)	1.4 (0.6-2.6)	<0.001
ADI (National); median (IQR)		63.0 (42.0-80.0)	72 (2)	53.0 (34.0-72.0)	66.0 (47.8-82.0)	70.0 (52.0-85.5)	<0.001
Outcome; n (%)	Started evaluation within 6 months of referral	2067 (46.1)	0 (0)	406 (22.8)	591 (41.3)	1070 (84.0)	<0.001

Abbreviations: AA, African American; ADI, Area Deprivation Index; BMI, Body Mass Index; IQR, interquartile range; Mi, miles; SBP, systolic blood pressure; SD, standard deviation; SVI, Social Vulnerability Index.

For Review Only

Table S6. Differences in Patient Characteristics by Race at Evaluation.

**Patient Characteristics**		**Overall, N=4471 (100%)**	**Overall Missing Data, N (%)**	**African American, N=1485 (33.2%)**	**Hispanic, N=1335 (29.9%)**	**White, N=1235 (27.6%)**	**Other/ Unknown N=416 (9.3%)**	**P-value**
Demographics								
Age (year); median (IQR)		56.1 (45.3-64.3)	0 (0)	54.5 (45.0-63.1)	53.3 (42.6-62.2)	60.0 (50.0-66.6)	56.6 (45.0-65.2)	<0.001
Sex; n (%)	Female	1801 (40.3)	0 (0)	687 (46.3)	528 (39.6)	453 (36.7)	133 (32.0)	<0.001
Marital Status; n (%)	Married	2673 (60.0)	13 (0)	722 (48.82)	827 (62.0)	821 (66.5)	303 (73.7)	<0.001
	Single	1785 (40.0)		757 (51.2)	507 (38.0)	413 (33.5)	108 (26.3)	
Smoking; n (%)	Active/Former	1523 (34.8)	99 (2)	474 (32.4)	417 (32.1)	516 (42.5)	116 (29.2)	<0.001
	Never	2849 (65.2)		987 (67.6)	883 (67.9)	697 (57.5)	282 (70.9)	
Preferred Language; n (%)	English	3940 (88.1)	1 (0)	1481 (99.7)	904 (67.7)	1218 (98.6)	337 (81.2)	<0.001
	Spanish	437 (9.8)		0 (0.0)	430 (32.2)	7 (0.6)	0 (0.0)	
	Other	93 (2.1)		4 (0.3)	1 (0.1)	10 (0.8)	78 (18.8)	
Employment; n (%)	Employed	1319 (30.1)	82 (2)	393 (26.9)	375 (28.5)	402 (33.3)	149 (36.8)	<0.001
	Unemployed	1950 (44.4)		705 (48.3)	719 (54.6)	387 (32.1)	139 (34.3)	
	Retired	1120 (25.5)		361 (24.7)	224 (17.0)	418 (34.6)	117 (28.9)	
Education; n (%)	Less than college	1619 (37.6)	170 (4)	455 (31.4)	746 (58.7)	315 (26.5)	103 (26.0)	<0.001
	College or higher	2682 (62.4)		992 (68.6)	525 (41.3)	872 (73.5)	293 (74.0)	
Insurance; n (%)	Medicare	2705 (60.5)	2 (0)	990 (66.7)	829 (62.1)	667 (54.1)	219 (52.8)	<0.001
	Medicaid	35 (0.8)		13 (0.9)	15 (1.1)	3 (0.2)	4 (1.0)	
	Private	1678 (37.6)		473 (31.9)	474 (35.5)	545 (44.2)	186 (44.8)	
	Other/None	51 (1.1)		9 (0.6)	17 (1.3)	19 (1.5)	6 (1.5)	
Clinical Characteristics								
BMI; n (%)	Underweight	68 (1.5)	23 (0)	19 (1.3)	18 (1.4)	19 (1.5)	12 (2.9)	<0.001
	Normal	1064 (23.9)		281 (19.0)	306 (23.0)	295 (24.0)	182 (44.4)	
	Overweight	1413 (31.8)		419 (28.3)	451 (34.0)	408 (33.1)	135 (33.0)	
	Obese	1903 (42.8)		760 (51.4)	553 (41.6)	509 (41.4)	81 (19.8)	
Hypertension; n (%)		2031 (45.4)	0 (0)	807 (54.3)	571 (42.8)	480 (38.9)	173 (41.6)	<0.001
SBP; median (IQR)		141.00 (125.0-159.0)	82 (2)	142.0 (125.5-160.0)	142.0 (126.0-161.0)	140.0 (123.0-157.0)	140.0 (127.0-159.3)	0.01
Diabetes; n (%)		2378 (53.2)	0 (0)	783 (52.7)	822 (61.6)	567 (45.9)	206 (49.5)	<0.001
Coronary Artery Disease; n (%)		1085 (24.3)	0 (0)	342 (23.0)	300 (22.5)	361 (29.2)	82 (19.7)	<0.001
Cerebrovascular Accident; n (%)		557 (12.5)	0 (0)	232 (15.6)	132 (10.0)	157 (12.7)	36 (8.6)	<0.001

For Review Only

Peripheral Vascular Disease; n (%)		786 (17.6)	0 (0)	296 (19.9)	224 (16.8)	215 (17.4)	51 (12.3)	0.002
Pulmonary Hypertension; n (%)		195 (4.4)	0 (0)	72 (4.9)	50 (3.8)	61 (4.9)	12 (2.9)	0.156
History of Malignancy; n (%)		252 (5.6)	0 (0)	75 (5.1)	40 (3.0)	122 (9.9)	15 (3.6)	<0.001
Dialysis Time (days); median (IQR)		366.0 (158.3-967.0)	2057 (46)	446.0 (175.5-1363.5)	365.0 (163.0-939.5)	297.5 (130.0-633.8)	281.0 (120.5-869.0)	<0.001
Dialysis Type; n (%)	Hemodialysis	2385 (98.8)	2057 (46)	921 (98.9)	810 (98.5)	474 (99.2)	180 (98.4)	0.701
	Peritoneal Dialysis	29 (1.2)		10 (1.1)	12 (1.5)	4 (0.8)	3 (1.6)	
Prior Transplant; n (%)		133 (3.0)	0 (0)	20 (1.4)	26 (2.0)	81 (6.6)	6 (1.4)	<0.001
Intended Donor; n (%)		1044 (23.4)	0 (0)	254 (17.1)	293 (22.0)	393 (31.8)	104 (25.0)	<0.001
Contextual-level Characteristics								
Distance to Transplant Center (Mi); median (IQR)		21.1 (12.6,55.6)	0 (0)	18.2 (11.2,34.7)	18.2 (11.2,34.7)	34.0 (19.3,89.7)	18.1 (13.8,25.8)	<0.001
Yearly Household Income ($); median (IQR)		63766.0 (46543.0-85444.0)	18 (1)	51765.5 (36222.0-69275.0)	51765.5 (36222.0-69275.0)	80913.50 (60864.0-104435.8)	101642.0 (73951.8-128333.0)	<0.001
Household Size; median (IQR)		2.9 (2.6-3.1)	13 (0)	2.93 (2.6-3.2)	2.93 (2.6-3.2)	2.8 (2.6-3.0)	3.0 (2.7,3.2)	<0.001
Monthly House Cost ($); median (IQR)		1187.0 (898.0-1481.0)	0 (0)	1152.0 (909.5-1400.0)	1152.0 (909.5-1400.0)	1224.5 (892.0-1525.0)	1481.0 (1195.0-1930.0)	<0.001
Commute Time to Health Center; median % (IQR)	<30 mins	26.7 (20.5-34.2)	0 (0)	26.4 (20.8-32.5)	26.4 (20.8-32.5)	27.0 (20.1-37.0)	23.6 (19.5-29.0)	<0.001
	30-45 mins	28.7 (24.4-32.7)		29.8 (26.8-33.7)	29.8 (26.8-33.7)	26.9 (19.8-30.7)	28.8 (26.9-33.7)	<0.001
	>45 mins	42.2 (34.5-48.6)		41.7 (34.8-47.3)	41.7 (34.8-47.3)	43.3 (32.5-52.0)	45.8 (38.7-51.4)	<0.001
Grandparents Responsible for Grandchildren; median % (IQR)		31.1 (21.3-41.8)	0 (0)	31.5 (21.9-45.3)	31.5 (21.9-45.3)	32.6 (21.2-45.4)	24.1 (16.6-33.5)	<0.001
Telework; median % (IQR)		6.9 (4.0-10.4)	0 (0)	5.8 (3.7-8.9)	5.8 (3.7-8.9)	8.3 (4.1-11.4)	10.4 (6.8-13.5)	<0.001
Own a Vehicle; median % (IQR)		98.3 (96.5-98.9)	0 (0)	97.9 (95.9-98.9)	97.9 (95.9-98.9)	98.5 (97.5-99.1)	98.5 (97.1-99.0)	<0.001

For Peer Review Only

Internet in Household; median % (IQR)		91.5 (84.7-95.5)	0 (0)	90.6 (83.8-95.2)	90.6 (83.8-95.2)	92.6 (87.4-96.6)	95.4 (91.8-97.4)	<0.001
Computer in Household; median % (IQR)		78.1 (65.6-87.5)	0 (0)	76.1 (63.5-85.7)	76.1 (63.5-85.7)	81.9 (71.3-90.9)	87.7 (81.5-94.5)	<0.001
Smartphone in Household; median % (IQR)		92.9 (88.2-95.6)	0 (0)	92.7 (87.4-95.5)	92.7 (87.4-95.5)	93.2 (89.3-95.9)	95.5 (93.1-97.1)	<0.001
Plumbing Facilities; mean median % (IQR)		98.9 (97.2-99.5)	0 (0)	98.9 (97.0-99.5)	98.9 (97.0-99.5)	98.7 (96.7-99.5)	99.5 (98.9-99.8)	<0.001
Census SSI; median % (IQR)		26.9 (13.9-38.4)	0 (0)	31.6 (18.9-41.3)	31.6 (18.9-41.3)	19.2 (11.1-31.3)	16.5 (9.7-27.9)	<0.001
SVI Below Poverty; median % (IQR)		21.9 (11.1-34.3)	14 (0)	25.1 (14.1-39.4)	25.1 (14.1-39.4)	16.1 (8.2-25.7)	11.8 (6.4-22.0)	<0.001
SVI Unemployment; median % (IQR)		25.7 (15.7-39.5)	14 (0)	29.6 (17.3-43.1)	29.6 (17.3-43.1)	21.7 (13.0-33.5)	25.2 (16.4-38.4)	<0.001
SVI Low Income; median % (IQR)		8.9 (6.1-12.6)	14 (0)	10.3 (7.4-14.2)	10.3 (7.4-14.2)	7.6 (5.3-10.6)	7.4 (5.0-11.5)	<0.001
SVI Single Parent; median % (IQR)		2.4 (1.3-3.9)	14 (0)	2.9 (1.7-4.6)	2.9 (1.7-4.6)	1.8 (0.9-3.2)	1.7 (0.9-3.1)	<0.001
SVI Mobile Homes; median % (IQR)		21.9 (11.1-34.3)	0 (0)	25.1 (14.1-39.4)	25.1 (14.1-39.4)	16.1 (8.2-25.7)	11.8 (6.4-22.3)	<0.001
SVI Crowding; median % (IQR)		1.2 (0.4-2.4)	14 (0)	1.3 (0.5-2.4)	1.3 (0.5-2.4)	0.8 (0.1-1.7)	0.8 (0.2-1.9)	<0.001
Outcome	Waitlisted within 12 months of evaluation	2073 (46.4)	0 (0)	564 (38.0)	564 (38.0)	704 (57.0)	217 (52.2)	<0.001

Abbreviations: BMI, Body Mass Index; IQR, interquartile range; Mi, Mile; SBP, systolic blood pressure; SD, standard deviation; SVI, Social Vulnerability Index.

For Review Only

Table S7. Patient Characteristics Predicting Risk of Dropping Out during Evaluation.
This table shows the characteristics of patients at low, middle, and high risk of dropout during evaluation before becoming waitlisted for a kidney transplant.

**Patient Characteristics**		**Overall, N=2235 (100%)**	**Overall Missing Data, N (%)**	**Low Risk, N=203 (9%)**	**Middle Risk, N= 1150 (51%)**	**High Risk, N=882 (39%)**	**P-value**
**Demographics**							
Age (year); median (IQR)		56.0 (45.3-64.3)	0 (0)	46.9 (36.8-55.2)	54.3 (44.5-62.6)	60.0 (50.1-67.3)	<0.001
Sex; n (%)	Female	891 (39.9)	0 (0)	69 (34.0)	459 (39.9)	363 (41.2)	0.171
Race/Ethnicity; n (%)	AA	743 (33.2)		34 (16.7)	313 (27.2)	396 (44.9)	<0.001
	Hispanic	667 (29.8)		33 (16.3)	355 (30.9)	279 (31.6)	
	White	617 (27.6)	0 (0)	99 (48.8)	356 (31.0)	162 (18.4)	
	Other/Unknown	208 (9.3)		37 (18.2)	126 (11.0)	45 (5.1)	
Marital Status; n (%)	Married	1323 (59.4)	7 (0)	147 (72.4)	753 (65.6)	423 (48.2)	<0.001
	Single	905 (40.6)		56 (27.6)	394 (34.4)	455 (51.8)	
Smoking; n (%)	Active or Former	756 (34.6)	50 (2.2)	41 (20.6)	346 (30.8)	369 (42.7)	<0.001
	Never or Unknown	1429 (65.4)		158 (79.4)	776 (69.2)	495 (57.3)	
Preferred Language; n (%)	English	1968 (88.1)	1 (0)	196 (96.6)	1017 (88.4)	755 (85.6)	<0.001
	Spanish	218 (9.8)		6 (3.0)	102 (8.9)	110 (12.5)	
	Other	48 (2.1)		1 (0.5)	31 (2.7)	16 (1.8)	
Employment; n (%)	Employed	674 (30.7)	36 (1)	160 (80.8)	419 (37.2)	95 (10.8)	<0.001
	Unemployed	976 (44.4		24 (12.1)	456 (40.5)	496 (56.6)	
	Retired	549 (25.0)		14 (7.1)	250 (22.2)	285 (32.5)	
Education; n (%)	Less than college	811 (37.7)	84 (4)	37 (18.4)	385 (34.6)	389 (46.4)	<0.001
	College or more	1340 (62.3)		164 (81.6)	727 (65.4)	449 (53.6)	
Insurance; n (%)	Medicare	1354 (60.6)	1 (0)	0 (0)	537 (46.7)	817 (92.6)	<0.001
	Medicaid	15 (0.7)		0 (0)	9 (0.8)	6 (0.7)	
	Private	842 (37.7)		203 (100)	589 (51.2)	50 (5.7)	
	Other/None	23 (1.0)		0 (0)	15 (1.3)	8 (0.9)	
**Clinical Characteristics**							
BMI; n (%)	Underweight	38 (1.7)	12 (1)	3 (1.5)	25 (2.2)	10 (1.1)	<0.001
	Normal	522 (23.5)		62 (30.7)	289 (25.3)	171 (19.5)	
	Overweight	708 (31.8)		65 (32.2)	377 (33.0)	266 (30.3)	
	Obese	955 (43.0)		72 (35.6)	451 (39.5)	432 (49.1)	
Hypertension; n (%)		1032 (46.2)	0 (0)	56 (27.6)	492 (42.8)	484 (54.9)	<0.001
SBP; median (IQR)		142.0 (124.0-160.0)	36 (2)	143.0 (127.0-156.0)	142.0 (122.0-160.0)	142.0 (124.0-160.0)	0.598
Diabetes; n (%)		1209 (54.1)	0 (0)	43 (21.2)	553 (48.1)	613 (69.5)	<0.001
Coronary Artery Disease; n (%)		533 (23.8)	0 (0)	6 (3.0)	206 (17.9)	321 (36.4)	<0.001

For Review Only

Cerebrovascular Accident; n (%)		286 (12.8)	0 (0)	5 (2.5)	105 (9.1)	176 (20.0)	<0.001
Peripheral Vascular Disease; n (%)		398 (17.8)	0 (0)	12 (5.9)	156 (13.6)	230 (26.1)	<0.001
Pulmonary Hypertension; n (%)		96 (4.3)	0 (0)	3 (1.5)	35 (3.0)	58 (6.6)	0.015
History of Malignancy; n (%)		118 (5.3)	0 (0)	9 (4.4)	52 (4.5)	57 (6.5)	0.143
**Contextual-level Characteristics**							
Distance to Transplant Center (Mi); median (IQR)		21.0 (12.6-51.0)	0 (0)	25.9 (17.7-43.7)	23.5 (13.7-76.5)	17.3 (11.0-32.7)	<0.001
Monthly Household Cost; median (IQR)		1165.0 (899.0-1481.0)	12 (1)	1653.0 (1257.0-1884.0)	1202.0 (913.0-1490.0)	1044.0 (880.5-1262.0)	<0.001
Commute Time to Health Center; median (IQR)	<30 mins	26.7 (20.7-34.2)	7 (0)	24.1 (16.7-29.8)	26.7 (20.5-35.6)	26.9 (22.6-33.1)	<0.001
Commute Time to Health Center; median (IQR)	30-45 mins	28.7 (24.1-32.8)	7 (0)	27.1 (21.8-30.0)	28.1 (23.5-32.1)	30.3 (26.6-33.8)	<0.001
Commute Time to Health Center; median (IQR)	>45mins	42.2 (34.8-48.6)	7 (0)	48.6 (41.3-57.0)	41.6 (33.1-48.6)	41.4 (35.5-45.8)	<0.001
Grandparents Responsible for Grandchildren; median (IQR)		31.1 (21.6-43.0)	17 (1)	25.3 (17.9-39.4)	30.4 (21.2-41.9)	31.9 (23.0-44.4)	<0.001
Telework; median (IQR)		6.8 (4.0-10.4)	9 (0)	11.9 (8.1-14.9)	7.1 (4.1-10.4)	5.7 (3.5-8.5)	<0.001
Own a Vehicle; median (IQR)		98.2 (96.5-98.9)	9 (0)	98.5 (97.6-99.2)	98.4 (96.7-99.0)	97.8 (95.8-98.7)	<0.001
SSI; median (IQR)		27.4 (15.4-38.5)	10 (0)	11.4 (7.7-18.9)	24.9 (13.0-36.2)	32.6 (22.2-42.5)	<0.001
Internet Subscription in Household; median (IQR)		91.4 (84.4-95.5)	9 (0)	96.0 (91.8-97.3)	92.0 (85.5-96.0)	89.2 (82.6-93.4)	<0.001
Computer in Household-median (IQR)		77.7 (65.0-87.5)	9 (0)	90.8 (81.8-94.1)	80.0 (67.5-89.2)	71.8 (61.8-83.2)	<0.001
Smartphone in Household; median (IQR)		92.9 (88.0-95.5)	9 (0)	95.5 (92.7-97.1)	93.1 (88.6-95.7)	91.5 (87.0-94.3)	<0.001
Plumbing Facilities; median (IQR)		98.9 (97.2-99.5)	9 (0)	99.3 (97.9-99.7)	98.9 (97.3-99.5)	98.7 (96.7-99.4)	<0.001
SVI Below Poverty; median (IQR)		22.2 (11.5-35.1)	9 (0)	9.7 (5.3-18.4)	19.6 (10.4-31.7)	28.6 (18.2-40.8)	<0.001
SVI Unemployment; median (IQR)		2.6 (1.6-4.0)	9 (0)	2.2 (1.4-3.4)	2.5 (1.5-3.8)	3.0 (1.9-4.3)	<0.001

SVI Low Income; median (IQR)		9.2 (6.3-12.8)	9 (0)	6.5 (4.7-9.1)	8.7 (6.1-12.2)	10.2 (7.5-13.7)	<0.001
SVI Single Parent; median (IQR)		2.4 (1.3-4.0)	9 (0)	1.9 (0.8-3.0)	2.2 (1.1-3.7)	2.8 (1.6-4.5)	<0.001
SVI Mobile Homes; median (IQR)		0.3 (0.0-2.7)	9 (0)	0.0 (0.0-2.0)	0.3 (0.0-3.3)	0.3 (0.0-2.5)	0.072
SVI Crowding; median (IQR)		1.3 (0.4-2.5)	9 (0)	0.4 (0.0-1.2)	1.1 (0.3-2.2)	1.8 (0.8-3.0)	<0.001
ADI (National); median (IQR)		60.0 (39.0-78.0)	15 (1)	32.0 (19.0-50.0)	55.0 (36.8-75.0)	69.0 (55.0-84.0)	<0.001
Outcome; n (%)	Waitlisted within 12 months of evaluation	1199 (53.6)	0 (0)	40 (19.7)	536 (46.6)	623 (70.6)	<0.001

Abbreviations: AA-African American; ADI-Area Deprivation Index; BMI-Body Mass Index; IQR-interquartile range; Mi-miles; SBP-systolic blood pressure; SD-standard deviation; SSI-Supplemental Security Income; SVI-Social Vulnerability Index.

For Review Only

Table S8. Differences in Patient Characteristics by Race at Waitlisting.

Patient Characteristics		Overall n=2457 (100%)	Overall Missing Data- N (%)	African American n=711 (31.4%)	Hispanic n=710 (28.9%)	White n=799 (32.5%)	Other/ Unknown n=237 (9.6%)	P-value
**Demographics**								
Age (year); median (IQR)		54.8 (44.3,63.0)	0 (0)	54.1 (44.5,62.3)	51.2 (41.1,60.6)	52.2 (40.0-58.0)	54.0 (44.3,63.6)	<0.001
Sex; n (%)	Female	982 (40.0)		311 (43.7)	293 (41.3)	186 (25.8)	73 (30.8)	0.003
Marital Status; n (%)	Married	1593 (65.1)	11 (0)	383 (54.1)	467 (65.9)	247 (34.3)	178 (76.4)	<0.001
	Single	853 (34.9)		325 (45.9)	242 (34.1)	278 (38.6)	55 (23.6)	
Smoking; n (%)	Active/Former	693 (31.1)	225 (9)	176 (27.9)	170 (26.4)	288 (39.0)	59 (27.2)	<0.001
	Never	1539 (69.0)		456 (72.2)	474 (73.6)	451 (61.0)	158 (72.8)	
Preferred Language; n (%)	English	2193 (89.3)	2 (0)	710 (99.9)	501 (70.6)	361 (45.2)	194 (82.6)	<0.001
	Spanish	217 (8.8)			209 (29.4)	257 (32.2)		
	Other	45 (1.8)		1 (0.1)		79 (9.9)	41 (17.5)	
Employment; n (%)	Employed	840 (35.0)	59 (2)	210 (30.2)	240 (34.4)	219 (27.4)	104 (45.8)	<0.001
	Unemployed	992 (41.4)		314 (45.2)	342 (49.1)	55 (6.9)	76 (33.5)	
	Retired	566 (23.6)		171 (24.6)	115 (16.5)	93 (11.6)	47 (20.7)	
Education; n (%)	Less than college	863 (36.4)	89 (4)	206 (29.6)	383 (56.4)	398.50 (208.3-753.5)	65 (28.1)	<0.001
	College or higher	1505 (63.6)		489 (70.4)	296 (43.6)	286 (98.6)	166 (71.9)	
Insurance; n (%)	Medicare	1243 (50.6)	1 (0)	404 (56.8)	381 (53.7)	352 (44.1)	106 (44.9)	<0.001
	Medicaid	11 (0.5)		2 (0.3)	5 (0.7)	3 (0.4)	1 (0.4)	
	Private	1178 (48.0)		302 (42.5)	318 (44.8)	434 (54.3)	124 (52.5)	
	Other/None	24 (1.0)		3 (0.4)	6 (0.9)	10 (1.3)	5 (2.1)	
**Clinical Characteristics**								
BMI; n (%)	Underweight	35 (1.7)	357 (15)	10 (1.8)	10 (1.6)	9 (1.3)	6 (3.0)	
	Normal	508 (24.2)		87 (15.3)	151 (24.8)	186 (25.8)	84 (41.8)	
	Overweight	721 (34.3)		192 (33.7)	209 (34.3)	247 (34.3)	73 (36.3)	
	Obese	836 (39.8)		280 (49.2)	240 (39.3)	278 (38.6)	38 (18.9)	<0.001
Hypertension; n (%)		1562 (63.6)		483 (67.9)	446 (62.8)	486 (60.8)	147 (62.0)	<0.001
SBP; median (IQR)		140.0 (124.0-156.0)	433 (18)	141.0 (125.0-157.0)	140.0 (124.0-157.0)	138.0 (122.0-156.0)	143.0 (127.0-156.3)	0.074
Diabetes; n (%)		1211 (49.3)	0 (0)	355 (49.9)	388 (54.7)	361 (45.2)	107 (45.2)	0.002
Coronary Artery Disease; n (%)		651 (26.5)	0 (0)	172 (24.2)	175 (24.7)	257 (32.2)	47 (19.8)	0.002
Cerebrovascular Accident; n (%)		235 (9.6)	0 (0)	70 (9.9)	65 (9.2)	79 (9.9)	21 (8.9)	0.933
Peripheral Vascular Disease; n (%)		619 (25.2)	0 (0)	183 (25.7)	172 (24.2)	219 (27.4)	45 (19.0)	0.060

For Review Only

Pulmonary Hypertension; n (%)		134 (5.5)	0 (0)	35 (4.9)	39 (5.5)	55 (6.9)	5 (2.1)	0.034
History of Malignancy (yes); n (%)		171 (7.0)	0 (0)	43 (6.1)	29 (4.1)	93 (11.6)	6 (2.5)	<0.001
Dialysis Time (days); median (IQR)		526.0 (285.0-1039.5)	1282 (52)	624.0 (348.0-1202.0)	558.50 (287.75-1085.0)	398.50 (208.3-753.5)	497.00 (228.50-846.50)	<0.001
Dialysis Type; n (%)	Hemodialysis	1155 (98.3)	0 (0)	387 (98.5)	388 (98.0)	286 (98.6)	94 (98.0)	0.902
	Peritoneal Dialysis	20 (1.7)		6 (1.5)	8 (2.02)	4 (1.4)	2 (2.1)	
Prior Transplant; n (%)		108 (4.4)	0 (0)	14 (2.0)	17 (2.4)	73 (9.1)	4 (1.7)	<0.001
Intended Donor; n (%)		873 (35.5)	0 (0)	209 (29.4)	242 (34.1)	338 (42.3)	84 (35.4)	<0.001
**Contextual-level Characteristics**								
Distance to Transplant Center (Mi); median (IQR)		21.6 (13.4-49.4)	0 (0)	32.8 (19.5-87.1)	19.6 (11.9-41.6)	19.6 (11.9-41.6)	18.3 (14.1-24.3)	<0.001
Yearly Household Income ($); median (IQR)		67245.0 (4940.50-92123.0)	9 (0)	83112.0 (64084.3-105157.0)	54516.0 (46543.0-71347.5)	54516.0 (46543.0-71347.5)	102060.0 (75033.0-131298.0)	<0.001
Household size; median (IQR)		2.9 (2.6-3.1)	7 (0)	2.8 (2.6-3.0)	3.0 (2.7-3.2)	3.0 (2.7-3.2)	3.00 (2.7-3.2)	<0.001
Monthly Household Cost ($); median (IQR)		1199.0 (915.0-1505.0)	9 (0)	1250.0 (915.0-1601.0)	1092.5 (877.0-1359.0)	1092.5 (877.0-1359.0)	1481.0 (1225.0-1950.0)	<0.001
Commute Time to Health Center; median % (IQR)	<30 mins	26.7 (20.4-34.2)	6 (0)	26.9 (20.2-36.8)	27.0 (22.9-34.5)	27.0 (22.9-34.5)	23.5 (19.4-28.1)	<0.001
	30-45 mins	28.7 (24.2-32.2)		26.8 (20.0-30.6)	28.9 (24.7-32.5)	28.9 (24.7-32.5)	28.8 (27.0-32.2)	<0.001
	>45 mins	42.8 (34.6-49.2)		43.9 (32.9-52.0)	41.4 (34.5-46.2)	41.4 (34.5-46.2)	46.0 (38.7-52.0)	<0.001
Grandparents Responsible for Grandchildren; median % (IQR)		30.4 (21.0-41.5)	13 (1)	31.5 (20.5-45.2)	31.1 (22.2-39.4)	31.1 (22.2-39.4)	24.1 (17.0-33.3)	<0.001
Telework; median % (IQR)		7.2 (4.3-10.8)	7(0)	8.6 (4.9-11.9)	6.4 (4.0-8.7)	6.4 (4.0-8.7)	10.4 (7.0-14.4)	<0.001
Own a Vehicle; median % (IQR)		98.3 (96.7-99.0)	7(0)	98.6 (97.6-99.1)	98.0 (96.3-98.9)	98.0 (96.3-98.9)	98.5 (97.1-98.9)	<0.001
Internet in Household; median % (IQR)		92.0 (85.1-96.0)	7(0)	93.1 (89.0-96.7)	89.9 (82.7-94.4)	89.9 (82.7-94.4)	95.4 (92.0-97.3)	<0.001
Computer in Household; median % (IQR)		80.0 (67.3-89.7)	7(0)	83.5 (72.9-91.0)	72.9 (61.8-84.6)	72.9 (61.8-84.6)	87.5 (82.3-94.4)	<0.001
Smartphone in Household; median % (IQR)		93.3 (89.1-95.7)	7(0)	93.6 (90.1-96.0)	92.2 (87.4-95.2)	92.2 (87.4-95.2)	95.5 (93.2-97.0)	<0.001

Plumbing Facilities; mean median % (IQR)		98.9 (97.3-99.6)	7(0)	98.9 (97.2-99.5)	98.7 (97.3-99.4)	98.7 (97.3-99.4)	99.5 (98.9-99.8)	<0.001
Census SSI; median % (IQR)		25.4 (12.2-36.5)	0 (0)	17.5 (10.1-28.7)	30.2 (19.7-40.4)	30.2 (19.7-40.4)	17.4 (10.1-27.4)	<0.001
SVI Below Poverty; median % (IQR)		20.3 (10.1-32.3)	7(0)	14.8 (7.8-24.1)	26.6 (16.1-38.9)	26.6 (16.1-38.9)	11.3 (6.4-20.3)	<0.001
SVI Unemployment; median % (IQR)		2.5 (1.5-3.8)	7(0)	2.2 (1.2-3.4)	2.5 (1.6-3.8)	2.5 (1.6-3.8)	2.5 (1.6-3.8)	<0.001
SVI Low Income; median % (IQR)		8.6 (5.9-12.0)	7(0)	7.5 (5.2-10.4)	9.1 (6.5-12.3)	9.1 (6.5-12.3)	7.4 (5.1-11.6)	<0.001
SVI Single Parent; median % (IQR)		2.4 (1.2-3.7)	7(0)	1.8 (0.9-3.1)	2.7 (1.5-4.0)	2.7 (1.5-4.0)	1.6 (0.8-3.0)	<0.001
SVI Mobile Homes; median % (IQR)		0.3 (0.0-2.9)	0 (0)	0.7 (0.0-6.6)	0.4 (0.0-2.6)	0.4 (0.0-2.6)	0.0 (0.0-0.6)	<0.001
SVI Crowding; median % (IQR)		1.1 (0.4-2.3)	7(0)	0.7 (0.1-1.7)	1.7 (0.8-3.0)	1.7 (0.8-3.0)	0.8 (0.2-1.9)	<0.001
Outcome	Completed transplant/still active on waitlist	763 (31.1)	0 (0)	270 (33.8)	223 (31.4)	223 (31.4)	53 (22.4)	0.010
	Death on waitlist	311 (12.7)		125 (15.6)	94 (13.2)	94 (13.2)	21 (8.9)	0.002

Abbreviations: BMI-Body Mass Index; IQR-interquartile range; Mi-Mile; SBP-systolic blood pressure; SD-standard deviation; SVI-Social Vulnerability Index.

For Review Only

Table S9. Patient Characteristics Predicting Waitlisting Outcomes.
This table shows the characteristics of patients at low-middle and high risk of dropout during waitlisting before receiving a kidney transplant.

Patient Characteristics		Overall* N=1228 (100%)	Overall Missing Data- N (%)	Low Risk N=647 (53%)	Middle Risk N= 538 (44%)	High Risk N= 43 (4%)	P-value
**Demographics**							
Age (year); median (IQR)		54.9 (44.5-63.0)	0 (0)	49.0 (39.0-59.0)	59.0 (50.7-65.4)	65.3 (56.8-69.7)	<0.001
Sex; n (%)	Female	481 (39.2)	0 (0)	260 (40.2)	209 (38.8)	12 (27.9)	0.274
Race/Ethnicity; n (%)	Hispanic	355 (28.9)	0 (0)	163 (25.2)	178 (33.1)	14 (32.6)	0.004
	AA	355 (28.9)		183 (28.3)	160 (29.7)	12 (27.9)	
	White	400 (32.6)		221 (34.2)	164 (30.5)	15 (34.9)	
	Other/ Unknown	118 (9.6)		80 (12.4)	36 (6.7)	2 (4.7)	
Marital Status; n (%)	Married	800 (65.2)	1 (0)	433 (66.9)	342 (63.7)	25 (58.1)	0.311
	Single	427 (34.8)		214 (33.1)	195 (36.3)	18 (41.9)	
Smoking; n (%)	Active or Former	351 (31.5)	115 (9)	149 (25.2)	182 (37.5)	20 (54.1)	<0.001
	Never or Unknown	762 (68.5)		442 (74.8)	303 (62.5)	17 (45.9)	
Preferred Language; n (%)	English	1097 (89.4)	1 (0.1)	588 (91.0)	471 (87.5)	38 (88.4)	0.291
	Spanish	108 (8.8)		48 (7.4)	55 (10.2)	5 (11.6)	
	Other	22 (1.8)		10 (1.5)	12 (2.2)	0 (0)	
Employment; n (%)	Employed	429 (35.8)	31 (3)	317 (50.5)	106 (20.1)	6 (14.3)	<0.001
	Unemployed	489 (40.9)		229 (36.5)	245 (46.5)	15 (35.7)	
	Retired	279 (23.3)		82 (13.1)	176 (33.4)	21 (50.0)	
Education; n (%)	Less than college	432 (36.5)	43 (4)	188 (29.5)	228 (44.5)	16 (44.4)	<0.001
	College or more	753 (63.5)		449 (70.5)	284 (55.5)	20 (55.6)	
Insurance; n (%)	Medicare	614 (50.0)	0 (0)	236 (36.5)	341 (63.4)	37 (86.0)	<0.001
	Medicaid	5 (0.4)		2 (0.3)	3 (0.6)	0 (0)	
	Private	598 (48.7)		402 (62.1)	190 (35.3)	6 (14.0)	
	Other/None	11 (0.9)		7 (1.1)	4 (0.7)	0 (0)	
**Clinical Characteristics**							
BMI; n (%)	Underweight	21 (2.0)	172 (14)	14 (2.5)	7 (1.5)	0 (0)	0.003
	Normal	248 (23.5)		153 (27.7)	90 (19.3)	5 (13.5)	
	Overweight	383 (36.3)		201 (36.4)	170 (36.4)	12 (32.4)	
	Obese	404 (38.3)		184 (33.3)	200 (42.8)	20 (54.1)	
Hypertension; n (%)		792 (64.5)	0 (0)	389 (60.1)	373 (69.3)	30 (69.8)	0.003
SBP; median (IQR)		141.0 (126.0-157.0)	207 (17)	143.0 (130.0-158.0)	139.0 (119.0-155.0)	132.0 (105.0-147.0)	0.628
Diabetes; n (%)		581 (47.3)	0 (0)	179 (27.7)	361 (67.1)	41 (95.3)	<0.001

For Review Only

Coronary Artery Disease; n (%)		323 (26.3)	0 (0)	84 (13.0)	212 (39.4)	27 (62.8)	<0.001
Cerebrovascular Accident; n (%)		115 (9.4)	0 (0)	35 (5.4)	74 (13.8)	6 (14.0)	<0.001
Peripheral Vascular Disease; n (%)		289 (23.5)	0 (0)	105 (16.2)	168 (31.2)	16 (37.2)	<0.001
Pulmonary Hypertension; n (%)		75 (6.1)	0 (0)	13 (2.0)	53 (9.9)	9 (20.9)	<0.001
History of Malignancy; n (%)		94 (7.7)	0 (0)	52 (8.0)	38 (7.1)	4 (9.3)	0.754
**Contextual-level Characteristics**							
Distance to Transplant Center (Mi); median (IQR)		21.7 (13.2-50.4)	1 (0)	22.2 (14.4-46.1)	20.5 (12.0-51.3)	27.1 (11.3-90.0)	0.066
Monthly Household Cost ($); median (IQR)		1189.0 (915.0-1490.0)	3 (0)	1292.0 (1028.0-1624.0)	1083.0 (879.0-1379.0)	837.5 (677.8-1100.5)	<0.001
Commute Time to Health Center; n (%)	<30 mins	26.7 (20.5-34.2)	2 (0)	26.4 (20.3-34.2)	27.0 (22.3-34.4)	27.7 (21.9-34.9)	0.201
	30-45 mins	28.6 (24.4-32.4)		28.5 (24.7-32.1)	28.9 (24.4-32.7)	28.0 (18.0-34.2)	0.412
	>45mins	42.3 (34.2-48.7)		44.6 (35.5-51.1)	41.2 (33.0-45.9)	38.3 (29.8-44.6)	<0.001
Grandparents Responsible for Grandchildren; n (%)		29.8 (20.9-41.3)	6 (0)	29.1 (19.5-39.2)	31.5 (22.2-41.6)	41.3 (27.4-60.9)	<0.001
Telework; n (%)		7.1 (4.3-10.7)	2 (0)	8.4 (5.2-11.9)	6.2 (3.8-9.0)	3.8 (2.8-8.2)	<0.001
Own a Vehicle; n (%)		98.3 (96.7-98.9)	2 (0)	98.4 (97.1-99.0)	98.0 (95.9-98.9)	97.3 (94.6-99.0)	<0.001
Internet Subscription in Household; mean (SD)		91.8 (85.4-95.7)	2 (0)	92.8 (88.2-96.5)	90.0 (83.5-94.6)	85.9 (77.8-92.8)	<0.001
Computer in Household-n (%)		79.9 (67.6-88.5)	2 (0)	83.1 (73.1-90.9)	73.2 (64.1-86.1)	65.0 (56.1-82.3)	<0.001
Smartphone in Household; n (%)		93.1 (89.1-95.7)	2 (0)	94.0 (91.0-96.3)	92.2 (87.3-95.0)	87.7 (82.8-94.1)	<0.001
Plumbing Facilities; n (%)		98.9 (68.9-100)	2 (0)	99.2 (83.3-100)	98.7 (78.2-100)	96.7 (68.9-100)	<0.001
SVI Below Poverty; mean (SD)		20.8 (10.3-32.2)	2 (0)	16.5 (8.1-26.8)	24.9 (14.2-36.5)	32.2 (19.6-45.7)	<0.001
SVI Unemployment; n (%)		2.5 (1.6-3.9)	2 (0)	2.4 (1.3-3.7)	2.9 (1.7-4.4)	3.5 (1.7-4.9)	<0.001
SVI Low Income; n (%)		8.5 (5.8-12.2)	2 (0)	7.8 (5.2-11.0)	9.6 (6.6-13.4)	10.2 (6.7-12.3)	<0.001
SVI Single Parent; n (%)		2.4 (1.2-3.8)	2 (0)	2.0 (1.0-3.6)	2.7 (1.4-4.1)	3.2 (1.7-4.0)	<0.001
SVI Mobile Homes; n (%)		0.3 (0.0-3.0)	2 (0)	0.3 (0.0-2.6)	0.3 (0.0-3.3)	0.7 (0.0-4.5)	0.150
SVI Crowding; mean (SD)		1.1 (0.3-2.2)	2 (0)	0.8 (0.2-1.9)	1.4 (0.6-2.8)	1.7 (0.8-3.4)	<0.001

For Review Only

ADI (National); median (IQR)		57.0 (36.0-75.0)	5 (0)	47.0 (30.0-67.0)	65.0 (44.0-79.0)	83.0 (58.0-92.0)	<0.001
Outcome; n (%)	Transplanted/ remained active on waiting list	381 (31.0)	0 (0)	105 (16.2)	246 (45.7)	30 (69.8)	<0.001

Abbreviations: AA-African American; ADI-Area Deprivation Index; BMI-Body Mass Index; IQR-interquartile range; Mi-miles; SBP-systolic blood pressure; SD-standard deviation; SVI-Social Vulnerability Index.

For Review Only

Table S10. Cox Proportional Hazards Model for Predictors of Waitlist Dropout

This analysis was performed as a supplementary analysis to account for the time-dependent outcome and variable follow-up duration in the waitlist cohort.

	Univariate		***Multivariate***	
	Hazard Ratio [95% CI]	p-value	Adjusted Hazard Ratio [95% CI]	p-value
Demographics				
Age				
0-44	[Reference]		[Reference]	
45-54	1.02 [0.7-1.48]	0.928	0.58 [0.23-1.45]	0.242
55-62	1.29 [0.92-1.82]	0.145	0.93 [0.39-2.22]	0.864
63+	1.83 [1.32-2.54]	< 0.001	0.68 [0.23-2.02]	0.489
Male Sex	1.19 [0.93-1.51]	0.162	0.62 [0.34-1.12]	0.115
Race / Ethnicity				
White	[Reference]		[Reference]	
Black	0.43 [0.31-0.57]	<0.001	0.51 [0.21-1.27]	0.147
Hispanic	0.45 [0.34-0.61]	<0.001	0.89 [0.23-3.44]	0.868
Other/unknown	0.48 [0.30-0.75]	<0.001	1.67 [0.76-3.69]	0.202
Currently Single	1.17 [0.91-1.51]	0.211	0.64 [0.34-1.23]	0.182
Historical/current smoker	1.36 [1.05-1.76]	0.022	1.10 [0.61-1.98]	0.745
Preferred language				
English	[Reference]		[Reference]	---
Spanish	0.82 [0.53-1.27]	0.735	0.95 [0.28-3.26]	0.78
Other/unknown	0.85 [0.35-2.08]	0.368	0.75 [0.1-5.8]	0.94
College Education	1.05 [0.82-1.36]	0.685	0.78 [0.43-1.42]	0.412
Employment				
Employed	[Reference]		[Reference]	---
Not Employed	1.36 [1.02-1.82]	0.039	1.05 [0.52-2.11]	0.897
Retired	1.64 [1.19-2.25]	0.002	0.66 [0.25-1.73]	0.399
Insurance				
Medicare	[Reference]		[Reference]	
Medicaid	0.23 [0.03-1.66]	0.146	0.67 [0.07-6.7]	
Private	0.85 [0.67-1.08]	0.177	0.39 [0.04-4.11]	
Other/none	1.06 [0.26-4.29]	0.933	---	---
BMI group				
Normal Weight	[Reference]		[Reference]	---

For Review Only

Underweight	0.24 [0.03-1.73]	0.157	---	---
Overweight	1.31 [0.94-1.83]	0.106	0.7 [0.33-1.51]	0.365
Obese	0.94 [0.67-1.31]	0.706	1.1 [0.53-2.28]	0.804
Systolic blood pressure				
<=120	[Reference]		[Reference]	
121-135	0.37 [0.26-0.53]	<0.001	0.35 [0.16-0.76]	0.008
136-150	0.28 [0.19-0.4]	<0.001	0.23 [0.1-0.54]	0.001
151-165	0.33 [0.23-0.49]	<0.001	0.42 [0.19-0.95]	0.037
166+	0.25 [0.17-0.39]	<0.001	0.13 [0.05-0.39]	<0.001
Clinical Characteristics				
Hypertension	1.14 [0.89-1.46]	0.294	1.03 [0.55-1.91]	0.93
Diabetes	1.22 [0.96-1.53]	0.106	1.55 [0.84-2.85]	0.16
Coronary Artery Disease	1.6 [1.26-2.04]	< 0.001	0.82 [0.44-1.52]	0.524
Peripheral Vascular Disease	1.28 [0.99-1.65]	0.054	0.77 [0.39-1.51]	0.446
Pulmonary Hypertension	2.37 [1.63-3.45]	<0.001	3.38 [1.34-8.54]	**0.01**
History of Malignancy	1.82 [1.25-2.67]	0.002	1.51 [0.61-3.74]	0.375
**Contextual-level Characteristics**				
Distance (miles)				
0-9	[Reference]		[Reference]	
10-14	0.68 [0.44-1.04]	0.078	1.43 [0.39-5.21]	0.587
15-19	0.54 [0.33-0.88]	0.013	0.28 [0.06-1.37]	0.116
20-24	0.69 [0.44-1.09]	0.1	0.43 [0.09-2.07]	0.295
25-44	0.99 [0.65-1.49]	0.945	1.31 [0.3-5.86]	0.72
45-119	1.18 [0.8-1.75]	0.409	5.02 [0.97-25.9]	0.054
120+	1.33 [0.89-1.99]	0.158	0.71 [0.15-3.35]	0.662
Monthly house cost ($)				
< 800	[Reference]	0.989	[Reference]	
800-999	0.84 [0.56-1.26]	0.401	0.81 [0.15-4.33]	0.812
1000-1199	0.91 [0.6-1.37]	0.638	0.34 [0.08-2.38]	0.343
1200-1399	0.93 [0.63-1.37]	0.716	0.88 [0.13-5.78]	0.878
1400+	0.95 [0.62-1.44]	0.801	0.81 [0.17-9.68]	0.805
< 30 minute commute				
0-20%	[Reference]		[Reference]	
20-25%	0.9 [0.62-1.31]	0.58	0.64 [0.15-2.8]	0.554
25-30%	0.96 [0.67-1.38]	0.835	0.55 [0.13-2.39]	0.425

30-40%	1.13 [0.78-1.62]	0.525	0.26 [0.04-1.97]	0.194
> 40%	1.4 [0.97-2]	0.069	0.23 [0.02-2.32]	0.215
30-45 minute commute				
0-25%	[Reference]		[Reference]	
25-30%	0.87 [0.65-1.17]	0.369	1.93 [0.53-7.02]	0.318
> 30%	0.85 [0.64-1.12]	0.261	1.38 [0.38-5.04]	0.621
>45 minute commute				
0-35%	[Reference]		[Reference]	
35-43%	0.69 [0.5-0.96]	0.025	0.72 [0.26-1.99]	0.529
43-50%	0.54 [0.38-0.77]	0.001	0.84 [0.21-3.28]	0.798
> 50%	0.73 [0.53-1]	0.053	0.55 [0.09-3.49]	0.525
Grandparents responsible				
0-20%	[Reference]		[Reference]	
20-30%	0.92 [0.64-1.33]	0.666	1.52 [0.51-4.53]	0.451
30-40%	1.28 [0.9-1.81]	0.163	0.92 [0.35-2.45]	0.875
> 40%	1.43 [1.03-1.98]	0.033	1.89 [0.66-5.39]	0.237
Work from Home				
0-4%	[Reference]		[Reference]	
4-7%	0.68 [0.48-0.96]	0.03	1.57 [0.59-4.15]	0.363
7-11%	0.81 [0.59-1.1]	0.179	1.56 [0.52-4.69]	0.427
> 11%	1.08 [0.78-1.49]	0.659	2.16 [0.5-9.21]	0.3
Owns Vehicle				
<97%	[Reference]		[Reference]	
97%-98.3%	0.89 [0.64-1.25]	0.502	0.44 [0.16-1.24]	0.122
98.3%-99%	0.83 [0.6-1.14]	0.252	0.34 [0.12-0.95]	0.04
>99%	1.17 [0.86-1.59]	0.328	0.82 [0.28-2.38]	0.71
Has internet connection				
<83%	[Reference]		[Reference]	
83%-90%	0.93 [0.64-1.33]	0.679	0.72 [0.16-3.27]	0.666
>90%	0.93 [0.66-1.3]	0.671	0.44 [0.08-2.49]	0.354
Has computer in home				
<80%	[Reference]		[Reference]	
80%-90%	0.94 [0.68-1.3]	0.707	0.85 [0.3-2.45]	0.769
>90%	0.92 [0.6-1.29]	0.626	3.61 [0.14-92.21]	0.437
Has smartphone				
< 89%	[Reference]		[Reference]	

89-93%	0.91 [0.65-1.28]	0.593	0.82 [0.26-2.58]	0.735
93-96%	0.93 [0.68-1.27]	0.65	1.09 [0.34-3.58]	0.881
>96%	0.91 [0.65-1.27]	0.572	1.32 [0.21-8.41]	0.769
Has plumbing				
< 97%	[Reference]		[Reference]	
97%-99%	1.12 [0.82-1.55]	0.478	4.16 [1.25-13.92]	0.021
99%-99.5%	0.93 [0.64-1.35]	0.702	4.64 [1.23-17.55]	0.024
>99.5%	1.01 [0.72-1.41]	0.958	9.87 [2.4-40.67]	0.002
SVI variables				
Poverty				
<1%	[Reference]		[Reference]	
1%-2%	0.83 [0.59-1.17]	0.292	0.74 [0.29-1.89]	0.528
2%-3%	0.9 [0.64-1.26]	0.541	0.65 [0.23-1.83]	0.412
>3%	0.91 [0.66-1.24]	0.533	0.52 [0.16-1.72]	0.287
Unemployment				
0-1.5%	[Reference]		[Reference]	
1.5%-2.5%	0.94 [0.68-1.29]	0.694	1.06 [0.49-2.3]	0.875
2.5%-4%	0.82 [0.59-1.13]	0.226	1.15 [0.52-2.55]	0.736
> 4%	0.89 [0.64-1.25]	0.508	0.79 [0.35-1.78]	0.57
Low Income				
0-6%	[Reference]		[Reference]	
6%-9%	0.99 [0.73-1.36]	0.993	0.75 [0.31-1.81]	0.515
9%-13%	0.95 [0.69-1.3]	0.729	1.97 [0.85-4.56]	0.114
>13%	0.79 [0.56-1.13]	0.197	0.72 [0.25-2.06]	0.538
Single Parent				
0-1.3%	[Reference]		[Reference]	---
1.3%-2.5%	0.92 [0.67-1.27]	0.623	0.68 [0.3-1.54]	0.357
2.5%-4%	0.89 [0.65-1.22]	0.482	0.55 [0.22-1.34]	0.192
> 4%	0.88 [0.63-1.24]	0.47	0.76 [0.32-1.81]	0.53
Mobile home				
0-0.01%	[Reference]		[Reference]	---
0.01%-3%	0.83 [0.63-1.1]	0.204	0.73 [0.36-1.51]	0.401
>3%	1.18 [0.89-1.56]	0.246	0.69 [0.26-1.83]	0.453
Crowding				
0-0.4%	[Reference]		[Reference]	---
0.4%-1.2%	0.84 [0.61-1.14]	0.264	2.11 [0.89-4.99]	0.088

For Review Only

1.2%-2.4%	0.68 [0.49-0.94]	0.019	1.47 [0.57-3.78]	0.425
>2.4%	0.78 [0.56-1.08]	0.134	1.85 [0.62-5.48]	0.267
National ADI rank				
0-34	[Reference]		[Reference]	---
35-54	0.83 [0.59-1.17]	0.292	2.14 [0.84-5.47]	0.11
55-74	0.92 [0.66-1.26]	0.594	2.13 [0.76-5.95]	0.148
75+	0.87 [0.62-1.2]	0.395	2.55 [0.77-8.49]	0.127

Abbreviations: AA-African American; ADI-Area Deprivation Index; BMI-Body Mass Index; CI-confidence interval; IQR-interquartile range; Mi-miles; SBP-systolic blood pressure; SD-standard deviation; SVI-Social Vulnerability Index.

For Review Only

Figure S1. Cumulative Hazard of Dropout After Waitlisting

Cumulative hazard of dropout over time among patients in the waitlist cohort. This analysis was performed as a supplementary sensitivity analysis using a Cox proportional hazards model to account for the time-dependent outcome and variable follow-up duration.

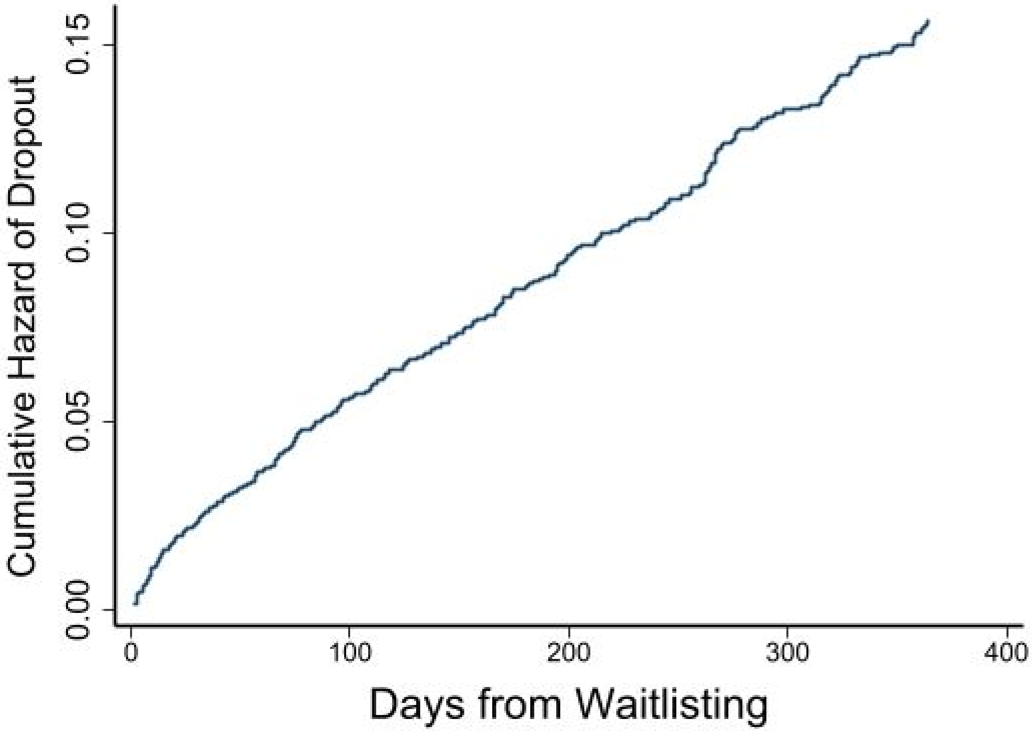

Only
